# New Parametric Model for Estimating the Viscosity of Choline Chloride-Based Deep Eutectic Solvents and Their Aqueous Mixtures

**DOI:** 10.3390/molecules31173003

**Published:** 2026-08-27

**Authors:** Salim Mokraoui, Irfan Wazeer, Lahssen El Blidi, Ihab Mohamed E. Koura, Mohamed K. Hadj-Kali

**Affiliations:** 1 Chemical Engineering Department, College of Engineering, King Saud University, P.O. Box 800, Riyadh 11421, Saudi Arabia; 2 Science Technology and Innovation Unit, King Saud University, P.O. Box 2454, Riyadh 11451, Saudi Arabia

**Keywords:** deep eutectic solvents, choline chloride, viscosity correlation, Grunberg–Nissan mixing rule, Arrhenius model

## Abstract

Deep eutectic solvents (DESs) derived from choline chloride (ChCl) have attracted significant interest as environmentally friendly alternatives to traditional solvents. However, their wider industrial application is often limited by high viscosity, which is strongly affected by temperature, water content, and the composition of hydrogen-bond donors (HBD). In this study, a new parametric correlation is presented for estimating the viscosity of aqueous ChCl-based DESs over broad composition and temperature ranges. The model combines the Arrhenius viscosity framework with the Grunberg–Nissan mixing rule, explicitly incorporating the effects of hydration and the HBA:HBD molar ratio through a four-parameter formulation. A comprehensive database of 1228 experimental viscosity measurements from various literature sources for eight chemically diverse aqueous ChCl-based DES systems, including monoethanolamine, ethylene glycol, glycerol, phenol, m-cresol, o-cresol, 1,2-propanediol, and 1,3-propanediol, was used for model development. The proposed correlation accurately reproduces experimental viscosities, achieving coefficients of determination between 0.990 and 1.000, with average absolute relative deviations ranging from 6.68% to 10.56%. Unlike existing models that require extensive, system-specific experimental matrices, this model effectively captures the substantial viscosity reduction due to hydration, the temperature dependence of viscous flow, and the influence of HBA:HBD stoichiometry utilizing only four global parameters per DES family based on molar composition.

## 1. Introduction

Deep eutectic solvents (DESs) have emerged as innovative green solvents, providing sustainable alternatives to traditional organic solvents and ionic liquids in various industrial applications. DESs consist of hydrogen-bond acceptors (HBAs) and donors (HBDs), demonstrating adjustable physicochemical properties, minimal toxicity, and biodegradability, which are consistent with the principles of green chemistry [1,2]. Choline chloride (ChCl)-based DESs have attracted considerable interest owing to their cost-effectiveness, straightforward synthesis, and adaptability in preparing eutectic mixtures with HBDs such as urea, glycerol, and organic acids [3,4]. Nonetheless, the elevated viscosity at ambient temperatures constitutes a significant constraint, especially for industrial processes that necessitate effective mass transfer, fluid management, and scalability [5]. The viscosity of DESs is affected by complex hydrogen-bonding networks, which are dependent on temperature, water content, and the molar ratio of HBA to HBD [6,7].

Aqueous DESs, resulting from the addition of water, effectively decrease viscosity while maintaining solvent functionality. This characteristic renders them appropriate for various applications, including enzymatic reactions, metal processing, and the extraction of bioactive compounds [8,9]. Alam et al. [10] demonstrated that a water content of 30–50% in ChCl-glycerol DES significantly enhanced the extraction yields of phenolic compounds from plant biomass by modulating the viscosity of the solvent, thereby underscoring the critical balance between viscosity reduction and solvent efficacy. Notwithstanding these advancements, the prediction of viscosity for aqueous DESs remains challenging owing to the nonlinear interactions among composition, temperature, and intermolecular forces. This situation emphasizes the need for the development of robust models suitable for industrial applications [11,12].

Recent advancements in computational methodologies have markedly improved our comprehension and predictive accuracy regarding the viscosity behavior of DESs. Molecular dynamics simulations have elucidated the microscopic mechanisms that govern viscosity in related systems, demonstrating how water molecules interfere with the hydrogen-bonding networks between HBA and HBD components, consequently diminishing intermolecular friction. Machine learning (ML) methodologies have demonstrated potential in correlating the composition of DESs with their physical properties. However, the majority of existing models are limited to specific families of DESs and necessitate comprehensive experimental validation [13]. Moreover, advanced viscosity reduction techniques beyond the addition of water have been developed, which include the integration of co-solvents such as alcohols and the formulation of ternary DESs with optimized component ratios [14,15]. The incorporation of these computational tools alongside experimental validation is facilitating the expedited rational design of next-generation DESs, characterized by tailored viscosity profiles suitable for various industrial applications [16].

The industrial uses of ChCl-based DESs encompass pharmaceuticals, biorefineries, food preservation, and metal processing. In the pharmaceutical industry, DESs function as effective mediums for the solubilization of drugs and the extraction of heat-sensitive active pharmaceutical ingredients. In biorefineries, DESs facilitate the pretreatment of lignocellulosic biomass by disrupting hydrogen bonds present in cellulose and hemicellulose, thereby enhancing the efficiency of enzymatic hydrolysis for biofuel production. Suopajärvi et al. [17] reported the successful delignification of wheat straw utilizing acidic DESs under mild conditions. The food industry employs DESs for the extraction of nutraceuticals, including polyphenols from orange peel waste. ChCl-based DESs have demonstrated superior yields in comparison to traditional solvents, while maintaining antioxidant activity [18]. In the domain of metal processing, ChCl-based DESs serve as alternatives to hazardous solvents utilized in electroplating and corrosion inhibition. Research has shown their efficacy in the recovery of precious metals from electronic waste [19]. These applications highlight the necessity for accurate viscosity models to enhance process parameters, equipment design, and energy consumption.

Despite their promising applications, scaling ChCl-based DESs to industrial production faces considerable economic challenges, primarily due to high solvent viscosity, which limits mass transfer rates and significantly increases pumping and mixing costs [20]. Reducing viscosity is therefore central to the economic viability of DES-based industrial processes. Ultimately, realizing the full market potential of DESs in areas such as carbon capture, energy storage, and pharmaceuticals will depend heavily on the development of standardized, reliable viscosity prediction models.

Current viscosity models for DESs, including the Arrhenius equation and the Vogel–Fulcher–Tammann–Hesse relationship, do not adequately represent the intricate behavior of aqueous systems [21,22]. The Arrhenius model, while offering satisfactory fits within limited temperature ranges, exhibits a tendency to underestimate viscosity at lower temperatures as a result of non-exponential dynamics [22]. ML methodologies have demonstrated considerable potential; however, they necessitate substantial datasets and frequently exhibit a deficiency in interpretability for engineering applications [13,23]. Recent meta-analyses conducted by Gygli et al. [24] have identified inconsistencies in the reporting of experimental data, noting that numerous studies fail to include measurement errors and provide insufficient details regarding DES desiccation methods, thereby complicating the process of model validation. Bakhtyari et al. [25] established a global correlation for 156 DESs, necessitating critical pressure and temperature alongside one reference viscosity, resulting in an average absolute relative deviation of approximately 10.4%. However, their model did not explicitly incorporate the plasticizing effect of water, which disrupts hydrogen bonds and modifies free volume dynamics. Lapeña et al. [21] indicated that the activation energy for viscous flow in ChCl-ethylene glycol diminishes with hydration, demonstrating the plasticizing effect of water that compromises the hydrogen-bond network. This behavior, in conjunction with the atypical viscosity–temperature trends observed for other aqueous DES systems such as reline, highlights the necessity to explicitly integrate water content and hydrogen-bond-donor-specific structural effects into predictive viscosity models.

Various modeling strategies have been developed to address these challenges. PC-SAFT, when combined with hydrogen-bonding models, has improved the accuracy of predictions for the thermodynamic properties of DES by explicitly accounting for association interactions [26]. Group contribution methods that use molecular descriptors have shown potential for estimating the physical properties of DES [27]. For viscosity, modified Eyring and VFT equations have enabled composition-dependent modeling for specific families of DES. Recently, Grunberg–Nissan mixing rules, integrated with the VFT framework, have been applied to both anhydrous and aqueous DES systems [28]. Molecular dynamics simulations and ML methods have shown strong accuracy for certain families of DESs, while the growing availability of standardized experimental databases is supporting further model development.

Advanced modeling approaches have improved our ability to predict DES characteristics, but substantial limitations remain. Most current models cannot describe the viscosity of aqueous DESs across wide composition and temperature ranges without significant experimental data specific to each system. To address this gap, this work introduces a four-parameter correlation to estimate the viscosity of aqueous ChCl-based DESs. The model explicitly accounts for the effects of hydration and HBA:HBD stoichiometry, combining the Arrhenius viscosity formalism with the Grunberg–Nissan mixing rule in a single, compact equation. The correlation is a valuable tool for solvent screening and process design in industrial applications, based on 1228 experimental data points from eight chemically distinct DES–water systems.

## 2. Results and Discussion

### 2.1. Fitted Parameters and Model Performance

The viscosity dataset from literature data was fitted into the developed model (Equation (9)) and the values derived from the parameters of the regression analysis are given in Table 1, together with the average absolute relative deviation (AARD) and the average relative deviation (ARD).

Table 1 reports fitted parameters and performance metrics for each system, with coefficients of determination from 0.990 to 1.000, AARD values from 6.68% to 10.56%, and ARD values from 2.11% to 5.99%. The positive ARD values indicate a slight overall tendency toward overestimation. Nevertheless, because the ARD values are considerably lower than the corresponding AARD values, the individual relative deviations also indicate that positive and negative deviations coexist and partially compensate each other.

For viscosity models of chemically diverse DES–water mixtures, that level of accuracy is generally good because the underlying fluids often show nonideal composition effects, sharp viscosity decay upon hydration, and strong temperature dependence [29]. The application domain of the proposed correlation (Equation (9)), including the valid ranges of temperature, water content, and HBA:HBD molar ratio, is clearly specified in Section 3.3. Additional iterative statistical parameter values have been provided in the Supplementary Material S2.

The model parameters were obtained through a global regression of all available data for each DES–water system rather than fitting individual literature datasets separately. As literature data contain some experimental variability, part of the model residuals may arise from differences in the original measurements rather than from the model itself. However, the high coefficients of determination (R^2^ = 0.990–1.000) and the low AARD values (6.68–10.56%) suggest that the variability between data sources is small and does not affect the overall trends captured by the proposed model.

The parameter trends are chemically informative rather than merely empirical. High *E*_0_ and *E*_1_ values can be interpreted as indicators of stronger activation-controlled resistance to flow, meaning the viscosity is more sensitive to temperature and composition because molecular rearrangement requires more disruption of the hydrogen-bond network.

The effective energetic parameter, *E*_0_, varies widely, from 3.74 kJ mol^−1^ for ChCl-1,3 Prop to 27.83 kJ mol^−1^ for ChCl-Glyc. This difference reflects variations in the energy required for viscous flow in the DES-rich region and is consistent with the different hydrogen bonding of the HBDs. The parameter *E*_1_, which is directly related to the HBA:HBD ratio, also varies considerably. The relatively high *E*_1_ values for the cresol and propanediol systems suggest that their flow behavior is more sensitive to DES composition within the studied range. However, these parameters should be considered effective model parameters rather than direct measures of hydrogen-bond strength, as they may also reflect changes in molecular association, and/or composition. The interaction parameter *a*_12_ is positive for all systems, ranging from 2.58 for ChCl-EG-W to 4.57 for ChCl-MEA-W, indicating a positive excess contribution to viscosity arising from water–DES interactions. Because this parameter is multiplied by *x*(1 − *x*), its effect is negligible at the pure-DES and pure-water limits and is strongest at intermediate water contents. The differences in *a*_12_ among the HBDs also show that this non-ideal behavior depends on the specific DES composition.

The differences in prediction accuracy among the investigated systems may be attributed to the following factors:

- The physicochemical characteristics of the DESs.

The investigated systems include a chemically diverse set of ChCl-based DESs with different intermolecular interactions and hydrogen-bonding networks. Systems with complex molecular interactions are expected to show larger deviations. For example, the ChCl-MEA-W, ChCl-Phenol-W, ChCl-oCres-W, and ChCl-1,2 Prop-W systems exhibit comparatively higher AARD values (9.3–10.56%) because of the nature and strength of hydrogen bonding and steric effects and aromatic HBD such as phenol, o-cresol, and m-cresol. MEA contains both hydroxyl and amino functional groups, leading to competing hydrogen-bond donor and acceptor interactions. Adjacent hydroxyl groups in 1,2 Prop create stronger associations and structural heterogeneity (greater than in 1,3 Prop). This is why such highly associated structures lead to larger deviations from relatively simple empirical expression. In contrast, the ChCl-Glyc-W system exhibits the lowest AARD (6.68%) because of its highly homogeneous and stable hydrogen-bond network. Similarly, the ChCl-G-W (7.73%), ChCl-mCres-W (7.61%), and ChCl-1,3 Prop-W (8.18%) systems are described with relatively good accuracy, probably because of their flexible molecular structure that produces a gradual change in hydrogen-bonding interaction as water content increases.

- The variable water content range among the investigated systems.

At high-water concentrations (>70 wt%), the hydrogen-bond network becomes disrupted and the DES transforms into an aqueous electrolyte. This transition may affect the molecular structure and result in increased deviations.

- The quantity and quality of experimental data.

This factor also influences the regression quality. Systems with extensive datasets with full temperature and composition domain, such as ChCl-Glyc and ChCl-EG, benefit from more statistically reliable parameter estimation.

- limitations due to the proposed correlation.

The model assumes that viscosity can be represented by a common functional form with four adjustable parameters, which may be insufficient to represent systems with more complex interactions and, hence, may lead to larger deviations.

**Figure 1:**
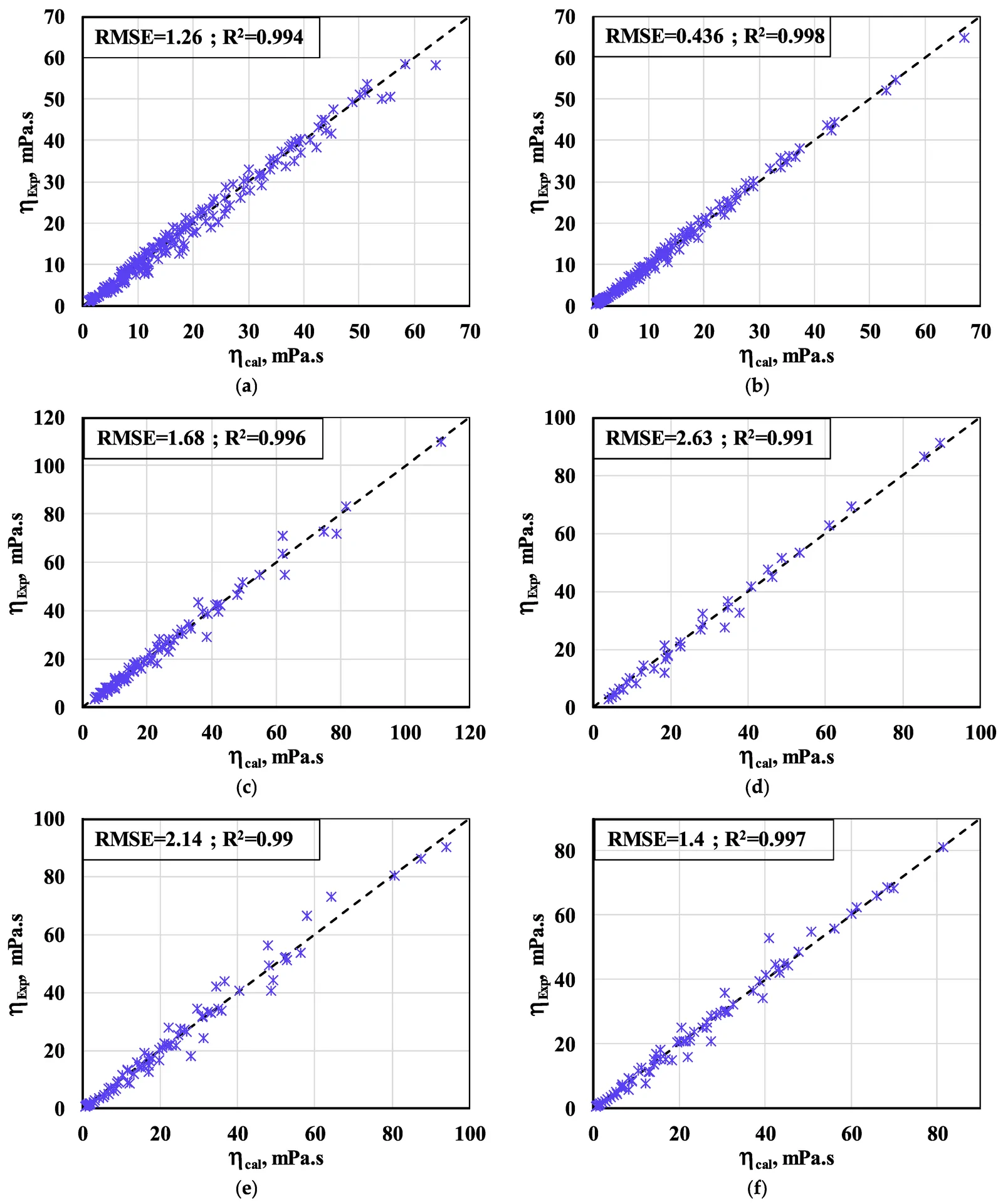

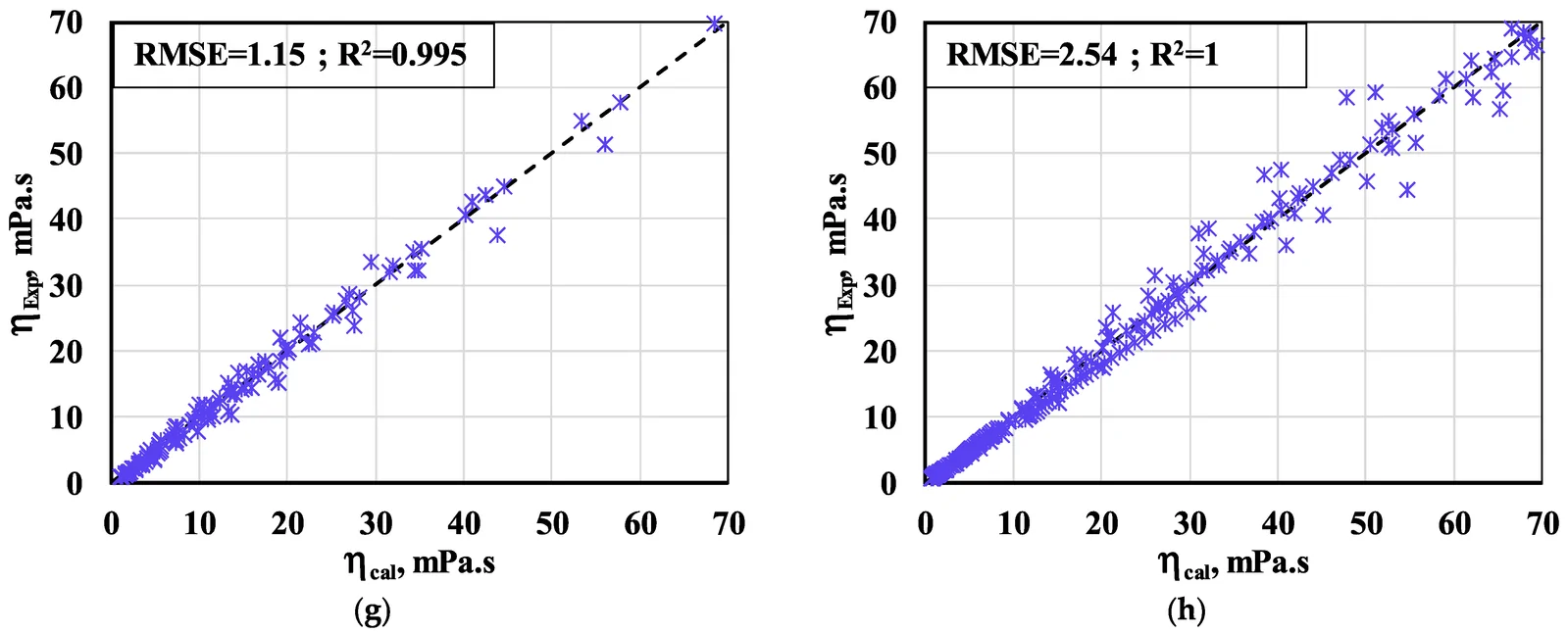
Experimental vs. calculated viscosity of the investigated systems: (**a**) ChCl-MEA-W; (**b**) ChCl-EG-W; (**c**) ChCl-mCres-W; (**d**) ChCl-oCres-W; (**e**) ChCl-1,2 Prop-W; (**f**) ChCl-1,3 Prop-W; (**g**) ChCl-Phenol-W; (**h**) ChCl-Glyc-W.

Figure 1 compares calculated and experimental viscosities for all eight systems. As displayed in Figure 1, the calculated viscosities show good agreement with the experimental data across all systems. Tight clustering around the ideal line suggests that the correlation captures the main temperature-composition-structure dependence, whereas broader spread at high viscosity would signal the common difficulty of modeling highly associated liquids where small structural changes produce large viscosity changes.

The eight parity plots imply that the chosen correlation is not merely overfitted to one fluid family but has reasonable generality across amine, glycol, glycerol, cresol, and phenolic DESs. This is significant because recent studies emphasize that DES viscosity prediction remains difficult, especially when water is added and the original supramolecular structure progressively transforms rather than disappearing abruptly [30].

Figure 1 also appears to show a common trend in DES modeling: prediction errors become larger at higher viscosities. This is expected because viscosity is highly sensitive to activation energy and hydrogen-bond interactions, so small structural changes can lead to much larger deviations in highly viscous mixtures [24].

### 2.2. Effect of Temperature and Water on Viscosity

**Figure 2:**
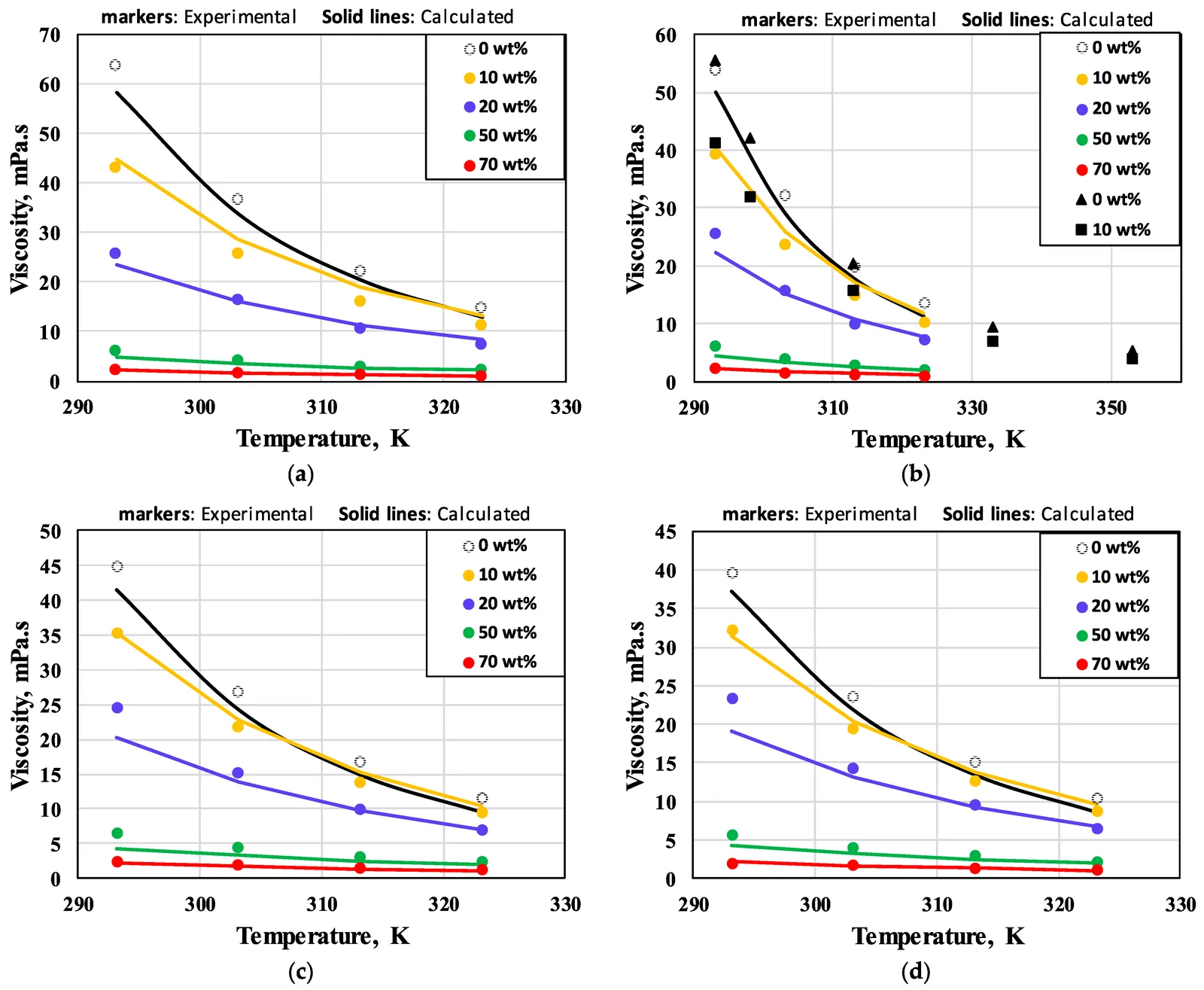
Effect of temperature and water content on the viscosity of different aqueous DES systems: (**a**) ChCl-MEA (1:5) + W; (**b**) ChCl-MEA (1:6) + W; (**c**) ChCl-MEA (1:8) + W (**d**) ChCl-MEA (1:10) + W [31,32].

**Figure 3:**
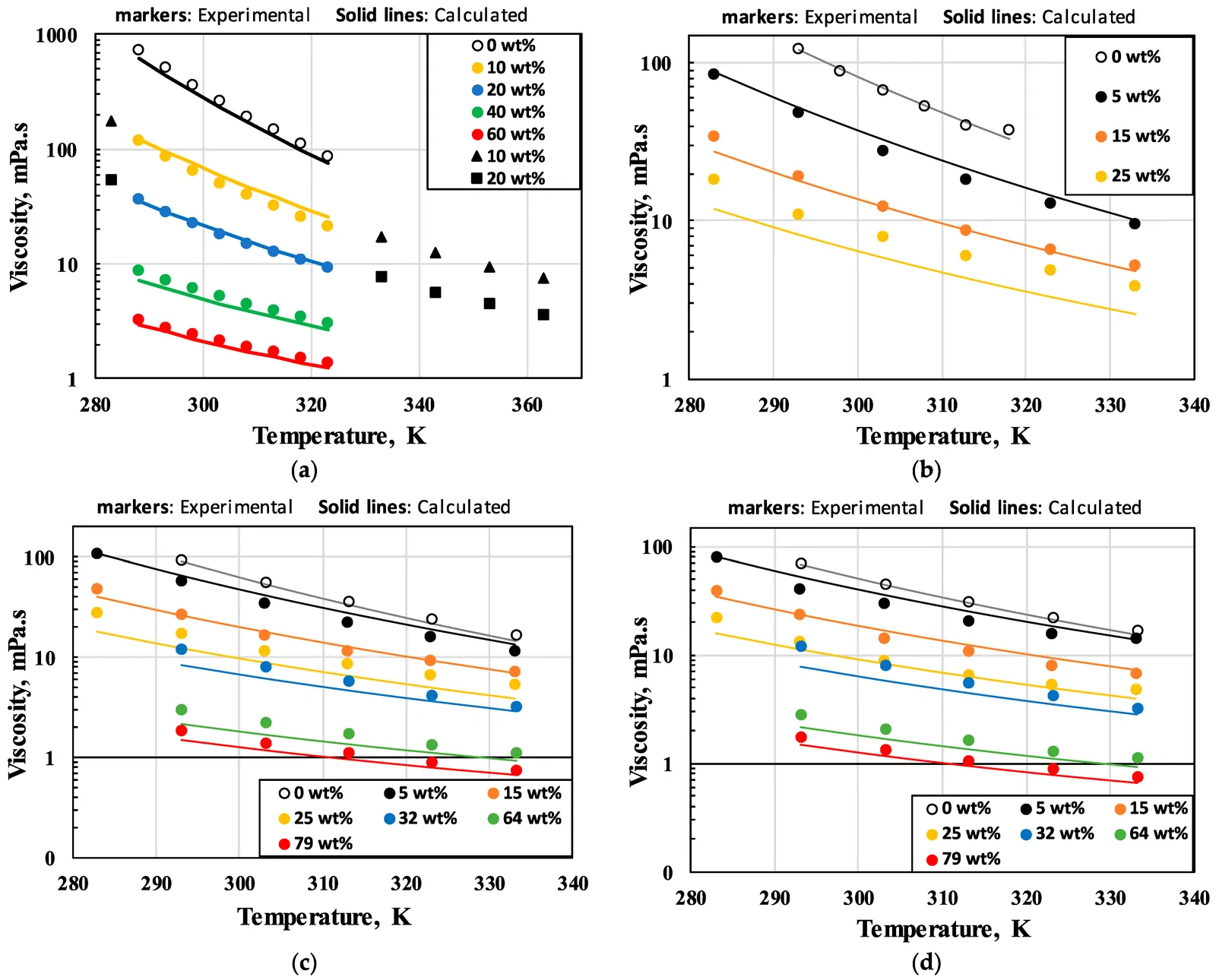
Effect of temperature and water content on the viscosity of different aqueous DES systems: (**a**) ChCl-Glyc (1:2) + W; (**b**) ChCl-oCres (1:3) + W; (**c**) ChCl-1,2 Prop (1:3) + W (**d**) ChCl-1,3 Prop (1:3) + W [28,34,35,36].

**Figure 4:**
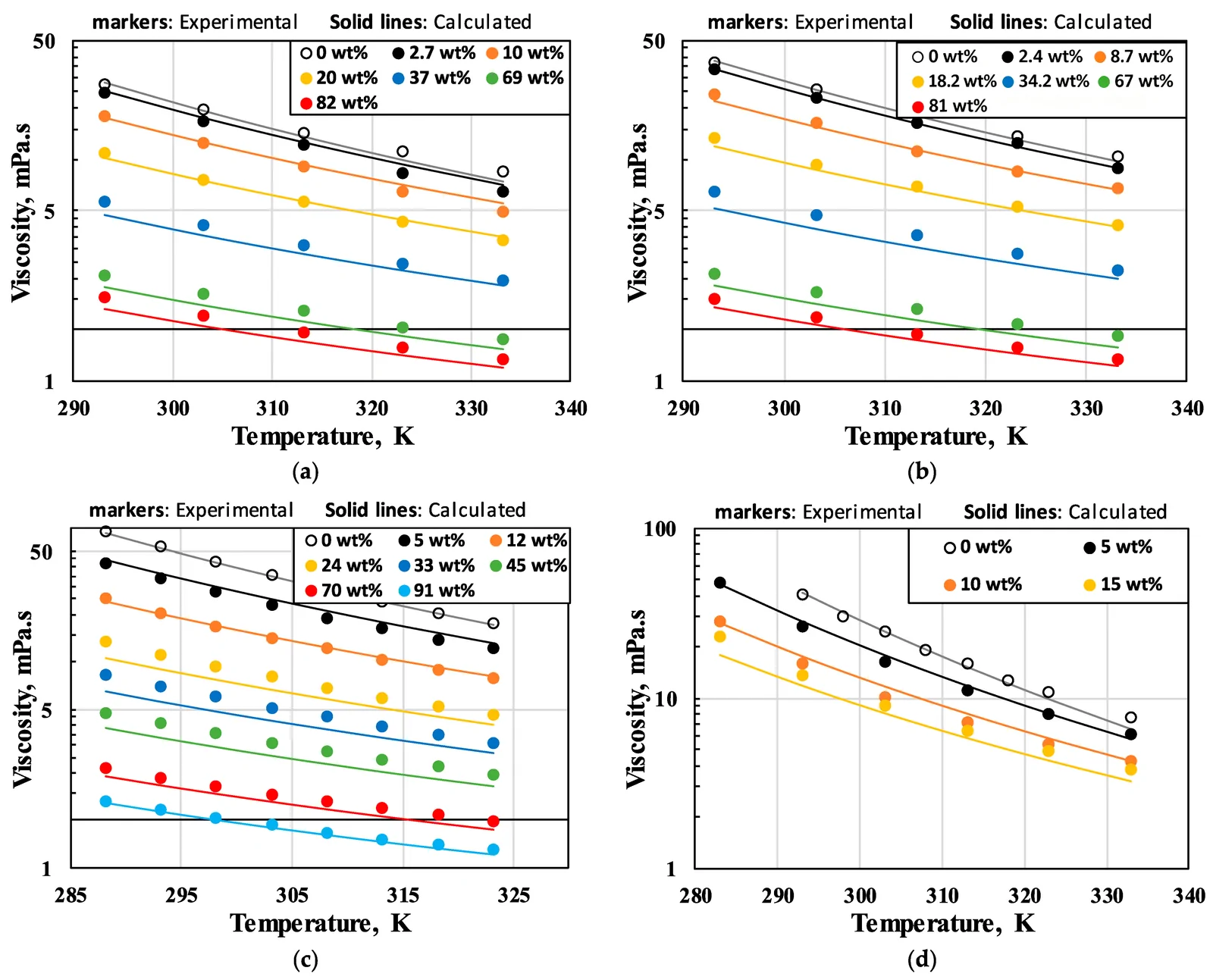

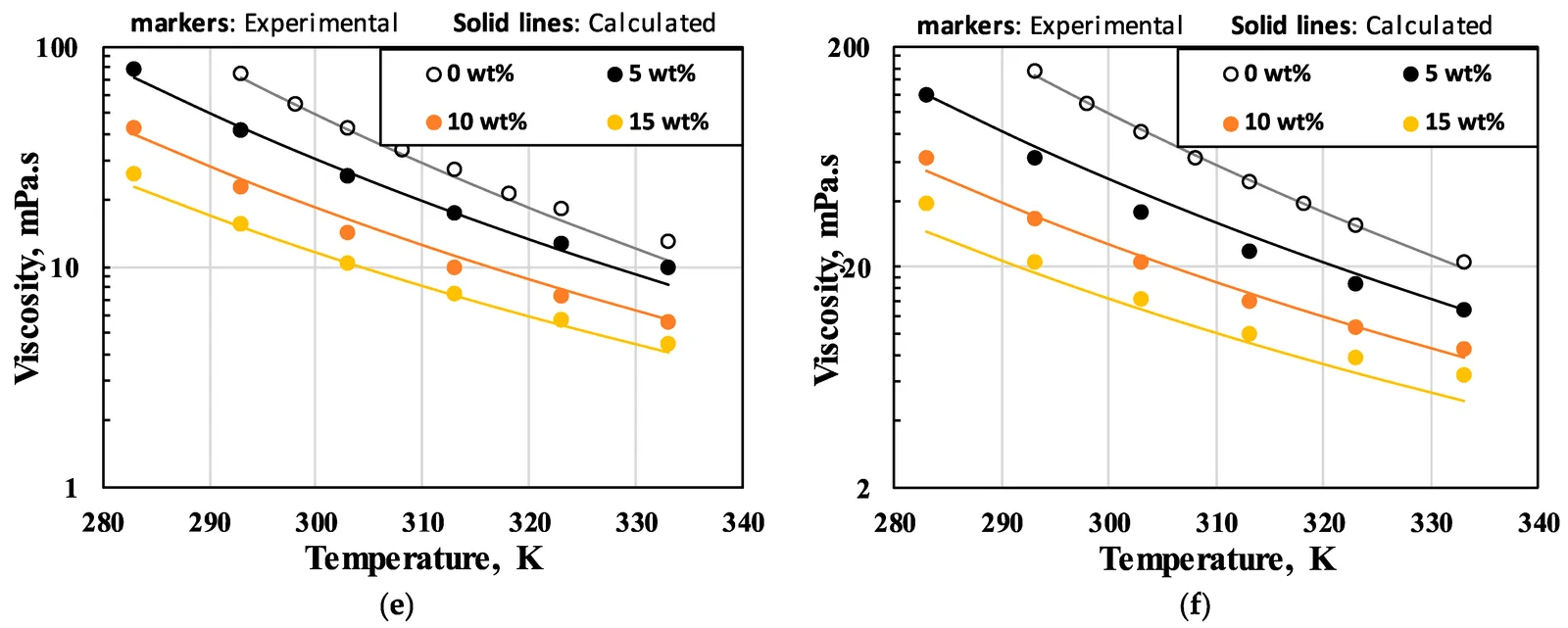
Effect of temperature and water content on the viscosity of different aqueous DES systems: (**a**) ChCl-EG (1:6) + W; (**b**) ChCl-EG (1:3) + W; (**c**) ChCl-EG (1:2) + W (**d**) ChCl-mCres (1:5) + W; (**e**) ChCl-mCres (1:3) + W; (**f**) ChCl-mCres (1:2) + W [28,35].

**Figure 5:**
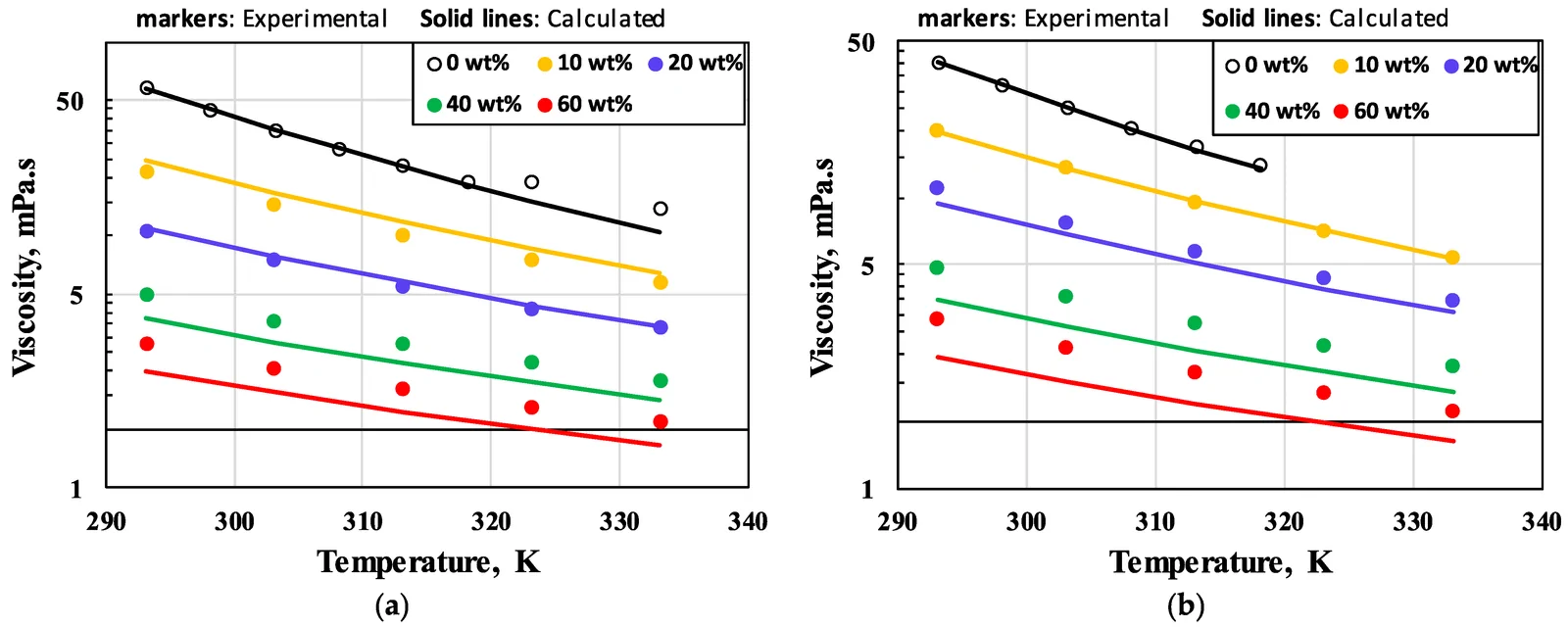
Effect of temperature and water content on the viscosity of different aqueous DES systems: (**a**) ChCl-Phenol (1:3) + W; (**b**) ChCl-Phenol (1:4) + W [38,39].

To illustrate the effects of temperature and water content on the viscosity of the systems listed in Table 1, the viscosities calculated using Equation (9) were compared with the corresponding experimental data, as presented in Figure 2, Figure 3, Figure 4 and Figure 5.

Across all these figures, the most immediate common observation is monotonic viscosity decay with increasing temperature and strong viscosity suppression with increasing water content, with the fitted solid lines closely tracking the experimental markers over the studied ranges. The figures also demonstrate the strong capability of the proposed model to accurately represent the experimental data across a broad range of temperatures, water contents, and HBA:HBD molar ratios.

Figure 2 presents the viscosity behavior of four ChCl-MEA mixtures with HBA:HBD molar ratios ranging from 1:5 to 1:10, each evaluated at water contents of 0, 10, 20, 50, and 70 wt%. In all cases, viscosity decreases markedly with increasing temperature. However, the most pronounced effect is the substantial reduction in viscosity observed with increasing water content. This behavior indicates that, within the investigated range, water content exerts a significantly greater influence on the system than temperature.

At fixed composition, the curves are distinctly non-linear with temperature, especially for 0 and 10 wt% water, which is typical of activated viscous flow in highly associated liquids [2], where strong hydrogen bonding produces high apparent activation barriers to flow and where dilution progressively lowers those barriers by increasing molecular mobility [5]. ChCl-MEA network remains strongly cohesive at low water content but undergoes substantial disruption once water content becomes high enough to compete effectively for chloride solvation and hydrogen-bonding sites, disrupting the DES network and reducing the strong interactions that cause high resistance to flow [24].

The effect of changing the MEA-rich ratio from 1:5 to 1:10 has a smaller effect on viscosity than adding water. The 1:6 and especially 1:8 mixtures show slightly higher viscosities than the 1:10 mixture over the same temperature range, indicating that excess MEA affects the structure and interactions within the network rather than causing a simple increase or decrease in viscosity with composition.

Figure 3 compares four different hydrogen-bond donors at the same graphical scale: glycerol, o-cresol, 1,2-propanediol, and 1,3-propanediol. Figure 3a has the broadest viscosity span, extending from values near 103 mPa·s at the low-temperature, dry end down to single-digit mPa·s at high temperature and high-water content on a logarithmic axis scale. This extreme range immediately identifies ChCl-Glyc as the most strongly associated liquid among the systems shown in Figure 2, Figure 3, Figure 4 and Figure 5. This is fully consistent with literature data, stating that glycerol-based DESs are highly viscous because glycerol forms strong and extensive hydrogen-bond networks that create strong intermolecular interactions [33]. The nearly parallel downward lines on the semilog plot indicate approximately exponential viscosity decrease with temperature within each water content. However, the much larger drop in viscosity as water content increases from 0 to 60 wt% indicates that adding water disrupts the glycerol-rich network far more effectively than moderate heating alone.

Figure 3b exhibits a much smaller composition set, limited to 0, 5, 15, and 25 wt% water. Figure 3b also shows that increasing both temperature and water reduces viscosity, and the dry system is markedly more viscous than the hydrated systems. Compared with glycerol-based DES, the absolute viscosities are lower. Because o-cresol contains only one hydroxyl group and a bulky hydrophobic aromatic ring, its DES structure should be less hydrogen-bonded than polyol-based systems and more influenced by aromatic interactions.

Figure 3c,d show a broad composition series up to 80 wt% water, with viscosity decreasing steadily as both temperature and water content increase. Relative to the glycerol system, all viscosity curves are lower, which is expected because replacing glycerol with a diol reduces the number of hydroxyl groups from three to two, resulting in a less connected hydrogen-bond network [24].

Figure 4 provides a useful comparison between a low-viscosity polyol-derived DES family (ethylene glycol-based) and an aromatic mono-phenolic family (m-cresol-based). The figure also demonstrates how well the model accommodates the experimental data within a wide water-content range in EG systems (0–91 wt%) and a narrow water-content interval in m-cresol systems (0–15 wt%).

Figure 4a–c cover ChCl-EG at 1:6, 1:3, and 1:2 ratios with water contents extending up to 82, 81, and 91 wt%, respectively. In all three cases, viscosity falls nearly linearly on the semilog scale with temperature and drops strongly as water increases, confirming that ethaline-like liquids are highly water-responsive [37].

Among all systems shown, the EG-based DESs exhibit some of the lowest viscosities at comparable temperatures and water contents. This agrees with literature data showing that ChCl-EG has a weaker and less interconnected hydrogen-bond network than ChCl-Glyc [37]. The very broad water content range in Figure 4a–c also demonstrates that the model remains stable as the system approaches water-rich compositions. That means the empirical viscosity expression (Equation (9)) succeeds for both DES-rich and water-rich regions.

Figure 4d–f cover ChCl-mCres at 1:5, 1:3, and 1:2 within 0–15 wt% water. The curves remain well separated even between 0, 5, 10, and 15 wt% water, showing that small water additions produce measurable viscosity reduction. However, the absolute effect of water is less pronounced than in glycerol-based or wider-range EG datasets, which is consistent with a system that does not possess the highly interconnected hydrogen-bond network characteristic of polyol-based DESs.

Figure 5a,b present viscosities of ChCl-Phenol at 1:3 and 1:4, each with 0, 10, 20, 40, and 60 wt% water. In both figures, viscosity decreases steadily with increasing temperature and drops progressively as water content increases from 0 to 60 wt%. The model lines closely follow the experimental points across the full composition range, with no obvious systematic deviation at any water content.

In contrast to cresol systems, which are limited to water concentrations below 25 wt%, phenol-based deep eutectic solvents remain miscible with water up to 60 wt%, covering a significantly wider compositional range. This is consistent with the greater hydrophilicity of phenol compared to m-cresol and o-cresol.

Figure 5a,b also indicates, notably, a nearly constant spacing between the different water contents. Thus, adding water to phenol systems reduces viscosity in a regular manner across the temperature range. Furthermore, the 1:3 system is slightly more viscous than the 1:4 system across the plotted temperatures, indicating that increasing the donor-rich environment, by adding further phenol, does not translate into stronger system hydrogen bonding.

Figure 2, Figure 3, Figure 4 and Figure 5 also imply that an aqueous DES should not be viewed as a simple binary dilution series. Recent studies of ChCl-based DESs emphasize microscopic heterogeneity, persistence of hydrogen-bonded clusters, and non-ideal mixing behavior. These factors help explain why viscosity remains strongly dependent on composition even after adding significant amounts of water [24].

The effectiveness of the new proposed model is clearly demonstrated in Figure 2, Figure 3, Figure 4 and Figure 5. The solid lines reproduce both the slope and the spacing of the experimental datasets across very different chemical families. The model successfully represents different behaviors across systems: strong temperature curvature in MEA systems, large viscosity ranges in glycerol systems, wide hydration effects in EG and propanediol systems, and comparatively narrower low-water ranges in cresol systems, generally without obvious systematic bias in most cases.

### 2.3. Effect of HBA:HBD Molar Ratio on Viscosity

**Figure 6:**
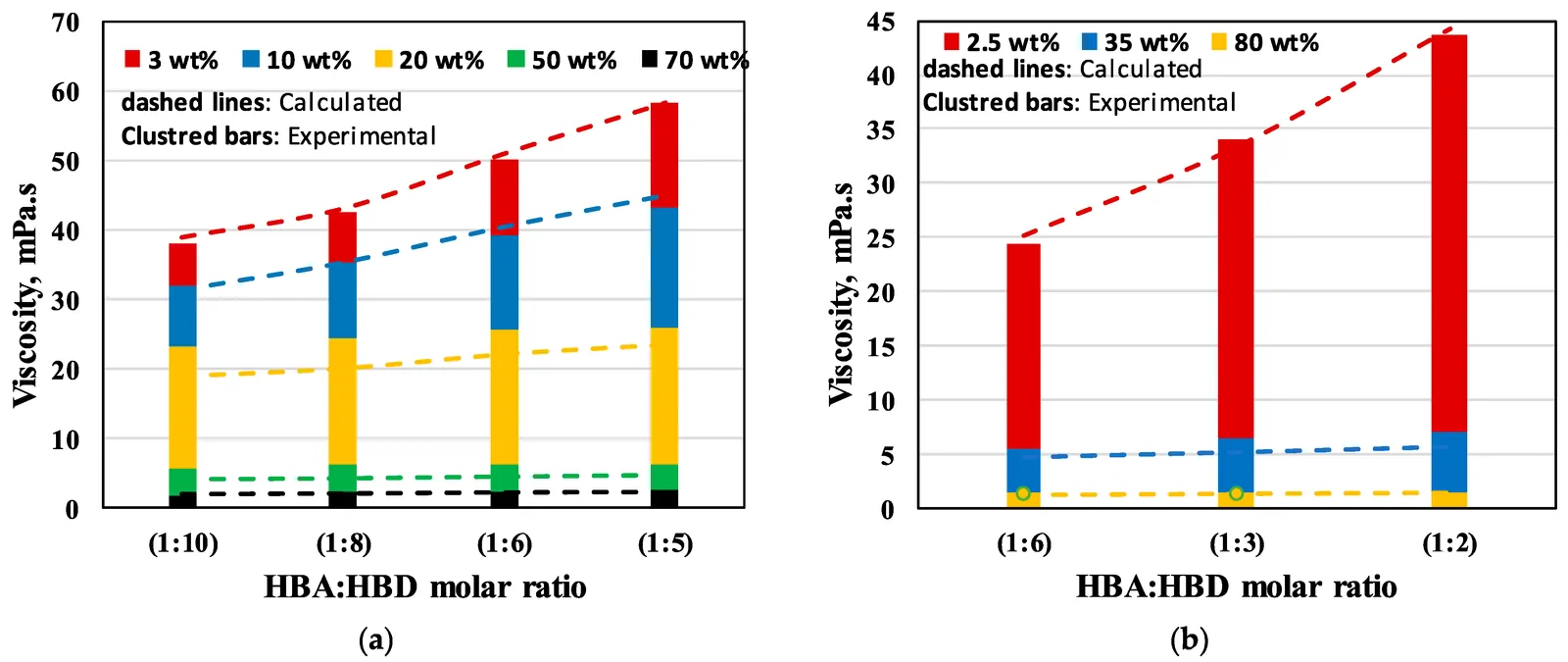

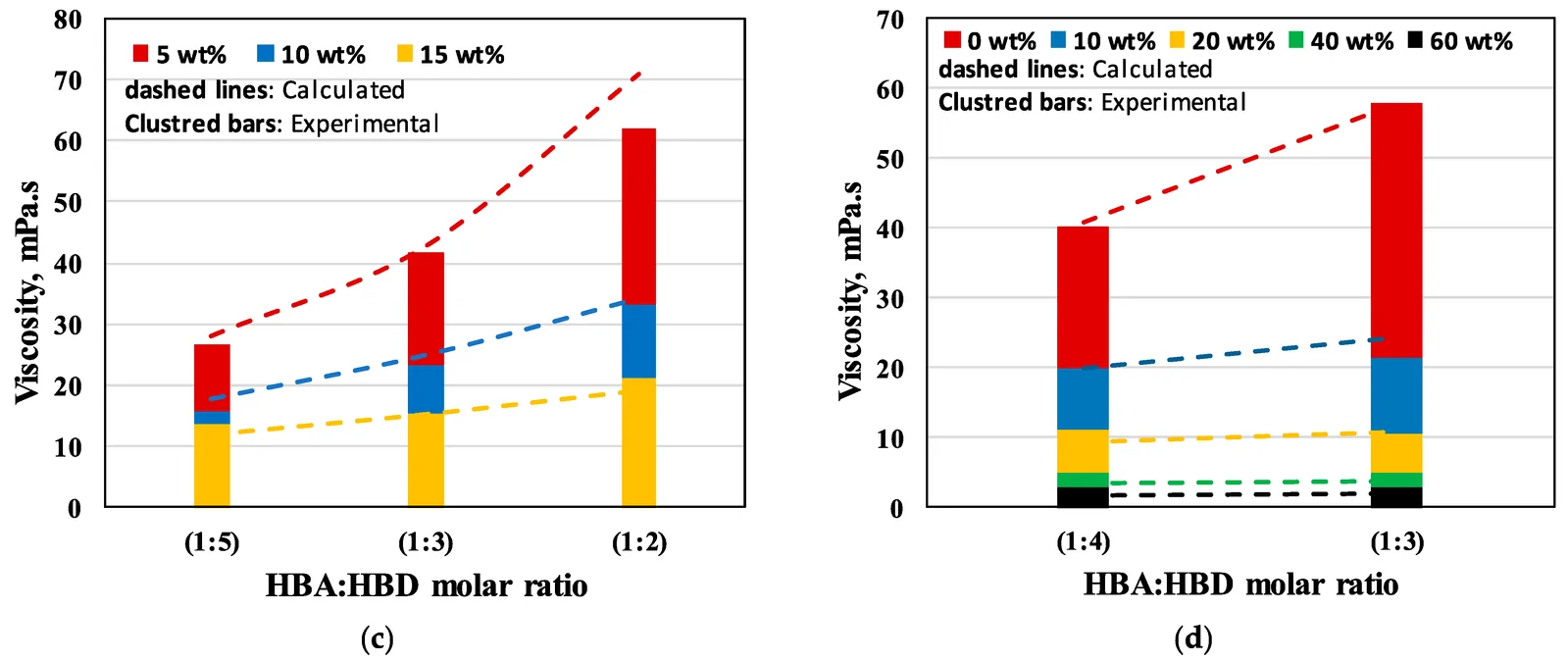
Effect of HBA:HBD molar ratio and water content on the viscosity of aqueous DES systems at 293 K: (**a**) ChCl-MEA-W; (**b**) ChCl-EG-W; (**c**) ChCl-mCres-W; (**d**) ChCl-Phenol-W [28,32,34,35,38,39].

Figure 6 plots the dynamic viscosity of ChCl-MEA-W, ChCl-EG-W, ChCl-mCresol-W, and ChCl-Phenol-W systems at 293 K against discrete HBA:HBD molar ratios, with the water content as a clustered-series variable. The following quantitative trends can be observed:

For each system, viscosity increases steadily as the HBA:HBD ratio rises because the activation barrier (*E*_0_ + *n E*_1_) grows linearly with *n*. When the system is rich in HBD, the hydrogen-bond network becomes most extensive, resulting in lower viscosity.

Within each ratio, viscosity decreases sharply with water content. The drop is highly non-linear, with the first 5–15 wt% of water reducing viscosity by 50–90% in aromatic-HBD systems (Figure 6c,d), confirming that water acts as a strong structure-breaker rather than a simple diluting agent.

The proposed model reproduces both effects with R^2^ ≥ 0.99 in every case (Table 1) and AARD between 6.68% (ChCl-glyc-W) and 10.56% (ChCl-1,2 Prop-W). The slightly larger AARD of the aromatic systems (10.1–10.3%) reflects both the smaller experimental databases (N = 36–122) and the higher sensitivity of viscosity to trace water in viscous, hydrogen-bonded liquids.

For ChCl-MEA-W system (Figure 6a), viscosities at 293 K span roughly 38 mPa·s at 1:10 up to 58 mPa·s at 1:5 for the same 3 wt% water content. Increasing water to 50 and 70 wt% collapses viscosity to less than 5 mPa·s, approaching that of MEA solutions. The fitted activation parameters (*E*_0_ = 21.2 kJ mol^−1^, *E*_1_ = 10.9 kJ mol^−1^) are larger than those of glycol-based analogs because the primary amine of MEA forms strong hydrogen bonds with choline, generating a more rigid network. This is consistent with Mjalli and co-workers, who identified MEA as one of the most viscous monoamine HBDs for choline-based DESs [40].

Ethaline-type systems (Figure 6b) display the lowest viscosities of the four panels because EG is a small, mono-functional H-bond donor. The viscosity of anhydrous 1:2 ChCl-EG at 293 K is reported at 36–45 mPa·s; the data clustered in Figure 6b at 2.5 wt% water lie between 24 (1:6) and 44 (1:2) mPa·s, which agrees with the results of Harifi-Mood and Buchner [41]. Above around 30 wt% water, the bars collapse to 4–6 mPa·s and the dashed curves predict an almost-flat response, exactly the saturation regime documented by [42] for ChCl-EG.

The ChCl-m-cresol-W system (Figure 6c) exhibits the strongest dependence on composition ratio. At 5 wt% water, viscosity rises from about 26 (1:5) to about 62 mPa·s (1:2). This system yields the largest fitted *E*_1_ value of the eight systems (20.1 kJ mol^−1^). The aromatic ring of m-cresol is responsible for the very high activation energy of viscous flow, as confirmed by Fan et al. [43]. Even modest water additions (5–15 wt%) reduce the viscosity by more than 50%, highlighting the strong sensitivity of these systems to hydration. This trend is consistent with the findings of Poe et al. [44], who reported that, for the ChCl-o-cresol system, minor water incorporation increased the self-diffusion coefficients by more than an order of magnitude, corresponding to a substantial decrease in viscosity. The dashed predictions in Figure 6c track the bars closely at 10 and 15 wt%, but slightly overestimate the 5 wt% data at 1:2. This is a known limitation of single-temperature parametrizations of aromatic DESs [44].

Figure 6d mainly reflects strong sensitivity to trace water and to the exact ChCl-phenol ratio. Adding 20 wt% water already brings viscosity to approximately 10 mPa·s. At 60 wt% the system is essentially aqueous (*η* ≈ 1–2 mPa·s). Picchio et al. [45] found that small mono-hydroxylated phenols give the lowest viscosity, which agrees with the present 1:4 molar ratio with 60 wt% water.

### 2.4. Comparative Performance Analysis

To evaluate the performance of the proposed correlation, it is compared with three representative models documented in the literature: the global correlation established by Bakhtyari et al. [25], the composition-dependent Eyring-based model developed by Mjalli and Naser [22], and the recent Grunberg–Nissan/VFT approach introduced by Peng et al. [28].

Bakhtyari et al. [25] formulated a comprehensive model encompassing 156 DESs with 1308 data points, resulting in an overall AARD of 10.4%. Although extensive in coverage, this model treats each HBA:HBD molar ratio as a separate pseudocomponent, requiring a reference viscosity measurement for each specific composition and, importantly, lacking the ability to accommodate aqueous DES systems. The AARD of the current model (6.68–10.56%) is comparable to that reported by Bakhtyari et al. [25] for the investigated ChCl-based systems. Furthermore, the present model achieves this while demonstrating applicability across a broad range of water contents (0–95 wt%).

Mjalli and Naser [22] employed a modified Eyring model to analyze nine ChCl-based DESs (non-aqueous), using 302 data points. They reported an overall ARD of 4.26%. Although their model demonstrates increased accuracy with non-aqueous DES data, it is limited to anhydrous conditions, requires 6 to 7 system-specific parameters to capture composition and temperature variations, and does not consider the plasticizing effect of water.

Peng et al. [28] introduced a Grunberg–Nissan/VFT framework applicable to ChCl-based DESs and their aqueous mixtures, resulting in AARD values of 2.95% for their regression set and 4.71% for their validation set for non-aqueous DESs. For aqueous mixtures, their Method 5 achieves reduced deviations; however, calibrating the system-specific parameters requires pre-existing experimental data across various temperatures and water contents for a fixed HBA:HBD ratio. This considerably increases the experimental workload for each new DES–water system. In contrast, the current model requires only four globally fitted adjustable parameters for each DES family, eliminating the need for extensive experimental matrices to predict viscosities at new compositions.

In summary, the proposed correlation model requires fewer parameters than existing models and does not need system-specific reference data, enhancing its practicality for solvent screening and process design when experimental data are limited.

## 3. Computational Models and Methods

### 3.1. Summary of Existing Models and Correlations

The viscosity of various types of DESs has been reported over a wide temperature range under atmospheric pressure; even though significant disparities may be observed, primarily due to diversities in experimental and preparation methods, as well as to the occurrence of some impurities [46].

Viscosity data of DESs are essential to design and optimize processes that use solvents, such as heat and mass transfer and separation processes [47,48]. Accurate knowledge of viscosity values is required to analyze and evaluate those various processes. Experimental viscosity data at specific conditions of temperature and composition are not always available, and thus, rapid estimation of the viscosity using models or correlations is highly suitable.

Various models have been developed to correlate DESs’ viscosity with temperature, composition, and molecular structure [29,49,50]. Among these models, traditional empirical correlations are widely applied and have proven to be highly valuable, offering both simplicity and practicality [25,51]. These correlations, typically temperature-dependent, are generally nonlinear equations with adjustable parameters obtained by fitting experimental data to the model [51]. Correlation-based models have the merits of being simple to use and can achieve high accuracy when sufficient experimental data are available. Hence, an effective empirical correlation requires an accurate, and in many cases, extensive experimental database.

For DESs and their co-solvent mixtures, the temperature dependence of viscosity is most commonly represented by the Arrhenius model [29,46,47,48,49,50,51,52,53] and the Vogel-Fulcher-Tammann (VFT) model [22,52,53,54].

(1) $$\mathrm{Arrhenius}\mathrm{model}:\;\eta =\eta_{0}\;exp(\frac{E_{a}}{RT})$$

where $\eta_{0}$ is the pre-exponential factor, and $E_{a}$ is the activation energy, both fitting parameters in

(2) $$\mathrm{VFT}\mathrm{model}:\;\ln\left(\eta \right)=A+\frac{B}{T-T_{0}}$$

A, B and T_0_ are adjustable parameters. T_0_ is known as the Vogel temperature.

Both correlations were found to be reliable for representing the temperature dependence of DESs’ viscosity [22,52,53,54]. While the Arrhenius model gives satisfactory fits for many DES types, the VFT model may result in superior results, particularly for more viscous DESs [51,55].

In dealing with empirical correlations, many studies have treated the DES as a pseudo-pure component, with the HBA:HBD molar ratio not explicitly included as a dependent variable. The other approach is to treat the DES as a binary mixture of two constituents (HBA and HBD), and hence, the molar composition is considered by the model formulation. Although the second approach is more realistic and consistent, both approaches are valid and yield an accurate description of viscosity [49,56].

In this study, aqueous DESs are treated as binary mixtures with their composition expressed in terms of water content given in mole fraction. The pseudo-pure DES molecular weight is calculated using the mole-fraction-weighted average, consistent with the convention adopted in all literature sources considered in this work. The corresponding molar composition formulation is provided in Supplementary Material S2. The viscosity of binary mixtures may be well estimated by applying an appropriate mixing rule [28,57].

### 3.2. The Proposed Correlation

The proposed model, presented hereafter, is based on the Arrhenius model [52] combined with the Grunberg–Nissan mixing rule [58]. To obtain a more practical model with minimum adjustable parameters, the aqueous DES mixture is regarded as a binary system (pure DES + water) with respect to the general expression of the mixture viscosity. However, the model formulation accounts implicitly for all components (HBA, HBD, water) by including the HBA:HBD molar ratio and the water molar fraction in the mixture.

According to the Grunberg–Nissan mixing rule the aqueous DES viscosity is expressed as

(3) $$\ln\eta =x_{1}\ln\eta_{1}+x_{2}\ln\eta_{2}+x_{1}x_{2}\;a_{12}$$

where indices 1 and 2 stand for DES and water components, respectively, and $a_{12}$ is the interaction parameter for the DES–water pair.

Using the fact that $x_{1}+x_{2}=1$, Equation (3) may be rearranged as

(4) $$\ln\eta =\ln\eta_{2}+(1-x_{2})\ln\frac{\eta_{1}}{\eta_{2}}+x_{1}x_{2}\;a_{12}$$

Which may be reduced to

(5) $$\eta =\eta_{2}\;{\left(\frac{\eta_{1}}{\eta_{2}}\right)}^{1-x_{2}}exp(x_{1}x_{2}\;a_{12})$$

According to the Arrhenius model, the viscosity of each component i, as function of temperature, is given by

(6) $$\eta_{i}=A_{i}\;exp(\frac{E_{i}}{R\;T})$$

where $A_{i}$ and $E_{i}$ are the pre-exponent factor and the activation energy for each component and R is the ideal gas constant.

Subsequently, the Arrhenius model suggests using an expression for the viscosities ratio $\frac{\eta_{1}}{\eta_{2}}$ with only two constants:

(7) $$\frac{\eta_{1}}{\eta_{2}}=A_{0}\;exp(\frac{E}{R\;T})$$

Equation (5) then becomes

(8) $$\eta =\eta_{2}\;A_{0}^{\left(1-x_{2}\right)}\;exp\left(\frac{E(1-x_{2})}{R\;T}\right)exp(x_{1}x_{2}\;a_{12})$$

Equation (8) includes three adjustable parameters: the pre-exponent factor *A*_0_, the activation energy E, and the interaction parameter $a_{12}$. Additionally, it includes the viscosity of pure water $\eta_{2}$ as a correcting factor. To feature the effect of the HBA to HBD molar ratio (*n*), Equation (8) has been primarily used for fitting a set of experimental data of different aqueous systems. For a given system, the fitting procedure has been repeated for different values of HBA:HBD molar ratios. Accordingly, a linear correlation has been identified for parameters E and *n*, whereas, for the other parameters, no significant trend was observed. Consequently, Equation (8) may be reformulated to obtain the following convenient model:

(9) $$\eta =\eta_{w}A_{0}^{\left(1-x\right)}\exp\left[\frac{\left(E_{0}+{nE}_{1})(1-x\right)}{RT}\right]\exp\left[\;x\left(1-x\right)a_{12}\right]$$

where $\eta_{w}$ is the viscosity of pure water, *x* denotes the water mole fraction, *n* is the HBA:HBD molar ratio, and *A*_0_, *E*_0_, *E*_1_, and $a_{12}$ are the model adjustable parameters.

Equation (9) highlights the viscosity dependence of the aqueous DES on both the temperature (*T*) and the molar composition (variables *x* and *n*).

This model can be viewed as a product of four physically distinct contributions: a water reference term, a composition-dependent prefactor, an activated-flow term, and a non-ideal mixing correction. This structure is consistent with the broader DES viscosity literature, where temperature dependence is often represented through activation-energy formulations and mixture effects are captured through excess-property or interaction terms [50].

The term $\eta_{w}$ provides the baseline viscosity at the operating temperature and anchors the whole correlation to the known behavior of pure water. The prefactor $A_{0}^{\left(1-x\right)}$ introduces a smooth adjustment that grows with DES content because (1−x) represents the non-water fraction. In Arrhenius-style transport-property models, prefactors are commonly associated with entropy-like, packing, or free-volume effects, so this term can be interpreted as accounting for structural and mobility changes that are not fully captured by the activation-energy part alone. As the DES fraction rises, the liquid generally becomes more structured and less mobile because of stronger ionic and hydrogen-bonding environments, and the prefactor provides a flexible way to represent this progressive departure from water-like flow behavior. The power-law dependence on (1−x) also ensures that the prefactor naturally tends toward unity as the system approaches pure water.

The exponential term $exp[(E_{0}+nE_{1})(1-x)/RT]$ has the form of a classical Arrhenius viscosity relation, where viscous flow is treated as a thermally activated process with an effective energy barrier. In that interpretation, $E_{0}$ is the base activation contribution associated with the DES framework, while $E_{1}$ adds a stoichiometry-sensitive contribution scaled by n, the HBA:HBD molar ratio. The factor $nE_{1}$ is physically reasonable because changing the HBA:HBD ratio alters the hydrogen-bond network, ion coordination environment, and molecular packing, all of which are known to influence DES viscosity strongly. Multiplication by (1−x) means this activation penalty increases as the DES fraction rises, while division by RT captures the well-established decrease in viscosity with increasing temperature.

The final factor $exp[x(1-x)a_{12}]$ is best interpreted as an excess interaction term that accounts for departures from ideal mixing between water and the DES. The product x(1−x) is widely used in mixture-property correlations because it is zero for pure water and pure DES but largest at intermediate compositions where non-ideal interactions are usually strongest. Physically, this term captures how water can either disrupt or reinforce the DES microstructure through hydrogen bonding, ion–dipole interactions, and changes in free volume. If $a_{12}$ is positive, the model predicts an upward deviation in viscosity at intermediate compositions, consistent with stronger transient structuring; if $a_{12}$ is negative, it represents a viscosity-lowering effect due to network disruption or enhanced mobility upon mixing.

The viscosity of pure water $\eta_{w}$ is a function of temperature and can be accurately estimated using several well-established correlations reported in the literature. In this study, the Vogel–Fulcher–Tammann equation [59] was selected to model the pure water viscosity:

(10) $$\eta_{w}=A\;exp\left(\frac{B}{T-C}\right)$$

where A = 0.02939 mPa.s, B = 507.88 K, and C = 149.3 K.

The developed model (Equation (9)) offers a significant advantage in that it requires only four adjustable parameters and considers the effect of both temperature and composition.

### 3.3. Experimental Database

Table 2 lists the aqueous DES systems used in this study along with the ranges of temperature, HBA:HBD molar ratio, water content and the corresponding references. These systems were selected due to the comprehensive experimental data available under various conditions.

Table 2 shows that the study spans a chemically diverse set of ChCl-based DESs, including amino alcohols (MEAs), polyols (EG, glycerol, 1,2-propanediol, 1,3-propanediol), phenolic donors (phenol, m-cresol, o-cresol), wide water ranges up to 95 wt%, and temperatures between 278 and 363 K. This breadth is important because DES viscosity is strongly affected by donor functionality, water dilution, and temperature, so a model tested across only one family would have limited transferability [51,63].

In the present study, the water content of all experimental data is expressed in mole fraction (mol%) as reported in the original literature sources, except for source [31], which reports the water content for the ChCl-MEA system in wt%. In this case, the water contents reported as wt% were converted to water mole fraction before regression. For practical purposes and to facilitate comparison and interpretation, the water content is presented as wt% in Table 2. Conversion formulas are provided in Supplementary Material S2. It should also be noted that, whenever an experimental water content was explicitly reported in the original source, the reported value was used directly. For nominally water-free DES samples for which no residual moisture measurement was reported, the composition was treated as (W = 0) wt%. This assumption may introduce a small uncertainty for hygroscopic DESs.

Table 2 also indicates that the viscosity database was compiled from several independent literature sources. Obviously, some variability associated with differences in experimental procedures cannot be completely excluded. Such differences may arise from the type of viscometer equipment, calibration procedures, temperature control and stability, sample preparation protocols, equilibrium time, and the purity of the DES constituents. In addition, slight variations in water content during sample handling may also influence the measured viscosity, particularly for aqueous DESs, whose viscosity is highly sensitive to composition. Since only peer-reviewed experimental data covering well-defined temperature, composition, and water-content ranges were considered, these effects were minimized.

A strong feature of the experimental matrix is that it deliberately includes both highly hydrophilic systems and more hydrophobic or strongly associating aromatic systems. That design allows the regression to probe whether a single functional form can capture very different extents of hydrogen-bonding and microstructural reorganization upon water addition, a central challenge emphasized in recent DES literature [30].

From a modeling perspective, ChCl-Glyc-W and ChCl-EG-W are especially valuable because they cover very broad water ranges, while the cresol-based systems challenge the model under restricted water miscibility conditions. Together, the eight systems create a balanced validation set in which both interpolation quality and robustness to different donor chemistries can be judged.

### 3.4. Fitting Procedure and Objective Function

The viscosity measurements across the eight DES + water systems listed in Table 2 were fitted to the model expressed by Equation (9). As stated earlier, this model expresses the viscosity as a function of temperature, HBA:HBD molar ratio, and water content.

For all those systems, a total dataset of 1228 experimental viscosity data points from literature was used to determine the model parameters and to assess the model performance. To achieve the nonlinear regression, most of the data collected from the literature sources were retained without deletion, as we believe that they resulted in satisfactory statistical regression performance. However, for the ChCl-Glyc system, some potential outliers were identified at high-water contents (>60 mol%). These data points were excluded based on an analysis of the residual estimates.

The fitting procedure was performed by using the fit functions available in MATLAB software (R2024a, MathWorks, Natick, MA, USA). The quality of the fitting was evaluated by minimizing the root mean square error, as an objective function, defined as

(11) $$RMSE=\sqrt{\frac{1}{N}\;\sum_{i=1}^{N}{\left(\eta_{i}^{exp}-\eta_{i}^{cal}\right)}^{2}}$$

## 4. Conclusions

A four-parameter viscosity correlation for aqueous choline chloride-based DESs has been developed by integrating the Arrhenius equation with the Grunberg–Nissan mixing rule. The model accounts for the effects of temperature, water content, and the molar ratio of hydrogen-bond acceptor to hydrogen-bond donor on the viscosity of aqueous choline chloride-based DESs systems. Parameters were fitted to 1228 experimental data points from eight choline chloride-based DES–water systems, yielding R^2^ values from 0.990 to 1.000 and average absolute relative deviations between 6.68% and 10.56%.

The results show that water is the primary factor in reducing viscosity, acting as a disruptor of the hydrogen-bond network rather than simply as a diluent. The ratio of hydrogen-bond acceptors to hydrogen-bond donors determines the baseline activation energy for viscous flow. The model reliably reproduces these effects across the glycol, glycerol, cresol, phenolic, and amino alcohol DES families, under conditions ranging from dry to 95 wt% water.

Although the proposed model shows good statistical performance, a formal sensitivity analysis of each parameter would provide further insight into the physical and practical significance of the correlation. Such a quantitative analysis could therefore be considered as a useful direction for future work. Unlike current correlations that require system-specific reference measurements or separate parameterization for each composition, this model needs only four parameters per DES family. This feature increases its practicality for solvent screening and process design when experimental data are limited.

While the current parameterization was developed for choline chloride-based DESs within the specified ranges (278–363 K, 0–95 wt% water, and HBA:HBD molar ratios of 1:2–1:10), the proposed framework is generally applicable. Because the functional form explicitly accounts for temperature, hydration, and HBA:HBD stoichiometry, it can be readily adapted to other DES families by refitting the four parameters once a limited set of experimental data is available. The favorable performance observed across five chemically distinct HBD classes (amines, glycols, glycerol, cresols, and phenols) supports the notion of transferability. The slightly larger deviations observed in MEA-, phenol-, and o-cresol-based systems indicate that additional refinement could improve descriptions of systems with particularly complex interactions. From an industrial perspective, the correlation can assist in the preliminary design of DES-based processes, such as extraction, CO_2_ capture, and metal processing, where viscosity affects pumping, mixing, and mass-transfer efficiency. Future research will focus on extending the model to include additional DES families and validating it with newly generated experimental data.

## Figures and Tables

**Table 1:** Parameters of the proposed correlation (Equation (9)) *.

DES + Water	N	*A*_0_	*E*_0_ (J.mol^−1^)	*E*_1_ (J.mol^−1^)	*a*_12_	R^2^	RMSE	AARD (%)	ARD (%)
ChCl-MEA-W	200	3.908 × 10^−3^	21,227	10,933	4.565	0.994	1.26	9.30	3.58
ChCl-EG-W	245	2.480 × 10^−1^	4573.7	10,746	2.580	0.998	0.436	7.73	5.88
ChCl-mCres-W	86	4.728 × 10^−3^	10,230	20,094	2.718	0.996	1.68	7.61	2.11
ChCl-oCres-W	36	5.362 × 10^−3^	12,448	19,588	2.988	0.991	2.63	10.13	5.99
ChCl-1,2 Prop-W	76	1.445 × 10^−2^	8862.2	18,338	3.727	0.990	2.14	10.56	5.02
ChCl-1,3 Prop-W	76	1.560 × 10^−1^	3741.7	13,573	3.718	0.997	1.4	8.18	3.69
ChCl-Phenol-W	122	2.133 × 10^−2^	15,854	10,239	3.177	0.995	1.15	10.29	5.43
ChCl-Glyc-W	387	1.749 × 10^−3^	27,833	4950.2	3.765	1.000	2.54	6.68	3.11

* MEA: monoethanolamine; EG: ethylene glycol; mCres: m-cresol; oCres: o-cresol; 1,2 Prop: 1,2-propanediol; 1,3 Prop: 1,3-propanediol; Glyc: glycerol; RMSE: root mean square error; $AARD=\frac{100}{N}\sum_{i=1}^{N}\left|\frac{\eta_{i}^{exp}-\eta_{i}^{cal}}{\eta_{i}^{exp}}\right|$; $ARD=\frac{100}{N}\sum_{i=1}^{N}\frac{\eta_{i}^{exp}-\eta_{i}^{cal}}{\eta_{i}^{exp}}.$

**Table 2:** Summary of investigated systems.

#	HBA	HBD	Mole Ratio (HBA:HBD)	Water Content, W (wt%)	Temperatures (K)	References
1	ChCl	MEA	1:5–1:10	0–70	293–353	[31,32]
2	ChCl	EG	1:2–1:6	1.2–95	288–333	[34,35]
3	ChCl	mCres	1:2–1:5	0–15	283–333	[28]
4	ChCl	oCres	1:3–1:5	0–25	283–333	[28]
5	ChCl	1,2 Prop	1:3–1:8	1–80	283–333	[28,35]
6	ChCl	1,3 Prop	1:3–1:6	1–80	283–333	[28,35]
7	ChCl	Phenol	1:2–1:6	0–60	293–333	[38,39]
8	ChCl	Glyc	1:2, 1:3	0–95	278–363	[34,36,60,61,62]

## Data Availability

The data presented in this study are available on request from the corresponding authors.

## Supplementary Materials

The following supporting information can be downloaded at https://www.mdpi.com/article/10.3390/molecules31173003/s1. Supplementary Materials S1: Definitions and composition convention. Supplementary Materials S2: Summary of Data Processing and MATLAB Implementation of the Nonlinear Regression Procedure. Supplementary Materials S3: Experimental data used in this study as reported in the original sources. Table S1. Viscosity data of ChCl-MEA-W (Ref. [31]). Table S2. Viscosity data of ChCl-MEA-W (Ref. [32]). Table S3. Viscosity data of ChCl-EG-W (Ref. [34]). Table S4. Viscosity data of ChCl-EG-W (Ref. [35]). Table S5. Viscosity data of ChCl-mCres-W (Ref. [28]). Table S6. Viscosity data of ChCl-oCres-W (Ref. [28]). Table S7. Viscosity data of ChCl-1,2 Prop-W (Ref. [28]). Table S8. Viscosity data of ChCl-1,2 Prop-W (Ref. [35]). Table S9. Viscosity data of ChCl-1,3 Prop-W (Ref. [28]). Table S10. Viscosity data of ChCl-1,3 Prop-W (Ref. [35]). Table S11. Viscosity data of ChCl-Phenol-W (Ref. [38]). Table S12. Viscosity data of ChCl-Phenol-W (Ref. [39]). Table S13. Viscosity data of ChCl-Glyc-W (Ref. [34]). Table S14. Viscosity data of ChCl-Glyc-W (Ref. [36]). Table S15. Viscosity data of ChCl-Glyc-W (Ref. [60]). Table S16. Viscosity data of ChCl-Glyc-W (Ref. [61]). Table S17. Viscosity data of ChCl-Glyc-W (Ref. [62]).

## Abbreviations

The following abbreviations are used in this manuscript:

1,2 Prop	1,2-Propanediol
1,3 Prop	1,3-Propanediol
AARD	Average absolute relative deviation
ChCl	Choline chloride
DESs	Deep eutectic solvents
EG	Ethylene glycol
Glyc	Glycerol
HBDs	Hydrogen-bond donors
HBAs	Hydrogen-bond acceptors
mCres	m-cresol
MEA	Monoethanolamine
ML	Machine learning
oCres	o-cresol
RMSE	Root mean square error
VFT	Vogel–Fulcher–Tamman
η	Dynamic viscosity of DES
$\eta_{w}$	Dynamic viscosity of pure water
R	Universal gas constant
x	Mole fraction of water
n	HBA:HBD molar ratio
A_0_	Pre-exponential model parameter
E_0_	Base activation energy parameter
E_1_	Composition-dependent activation energy parameter
$a_{12}$	Non-ideal interaction parameter
N	Number of experimental data points
