# Supplementary material for: New Parametric Model for Estimating the Viscosity of Choline Chloride-Based Deep Eutectic Solvents and Their Aqueous Mixtures
Source: Molecules. 2026 Aug 27;31(17):3003. doi: 10.3390/molecules31173003 (PMC13567486; doi:10.3390/molecules31173003)
Supplement: Supplementary file 1 [file molecules-31-03003-s001.zip › molecules-4476958-supplementary.pdf]

S1: Definitions and composition convention

For each DES, the HBA:HBD molar ratio was represented by the numerical parameter (n), defined as:

$$n = \frac{n_{HBA}}{n_{HBD}}$$

For example, for ChCl:EG (1:2), $n = 1/2$

The molecular weight of the pseudo-pure DES is calculated by the average molar mass using the mole fractions of each constituent component:

$$M_{DES} = x_{HBA}M_{HBA} + x_{HBD}M_{HBD}$$

For example, for ChCl:EG (1:2): $M_{DES} = \frac{1}{3}M_{ClCh} + \frac{2}{3}M_{EG}$

For experimental data reported in wt%, the corresponding water content was converted to mole fraction using the following equation:

$$x = \frac{W/M_w}{W/M_w + (100 - W)/M_{DES}}$$

Where M_w is the molar mass of water, and W is the water content in wt%

The reported ranges of W in Table 2 were obtained by converting the experimental water mole fractions provided in the literature to wt% using the following equation:

$$W = \frac{x_w M_w}{x_w M_w + (1 - x_w)M_{DES}} \times 100$$

S2: Summary of Data Processing and MATLAB Implementation of the Nonlinear Regression Procedure

The nonlinear regression procedure is performed by the following methodology:

Step 1:

For each DES-water system, raw viscosity data are extracted from literature sources. This includes, HBA:HBD molar ratio, composition or water content (mol% or wt%), temperature (K), and experimental viscosities (mPa.s). Composition conversions are performed whenever required as explained in S1. The data are stored in a matrix form for processing in MATLAB

Step 2:

The MATLAB routine is implemented by defining the model equation (Eq. 9) as a MATLAB function with 4 independent variables corresponding to the adjustable parameters (A_0, E_0, E_1, and a_{12})

Step 3:

The MATLAB fitnlm function was selected and employed with different robust weighting functions, including bisquare, cauchy, and huber functions. The use of robust weight functions allows to possibly reduce the large residuals during iterative least squares estimations due to by potentially anomalous literature measurements.

Step 4:

Starting values of the vector adjustable parameters are set to [1 1 1 1] as initial guess. However, sensitivity to initial guess is tested by running the regression from several different initial guess in order to assess robustness and consistency of convergences to the same approximate solution.

Step 5:

Define the following numerical optimization settings:

- Maximum iterations: 700
- Function tolerance: 10^{-10}
- Parameter-step tolerance: 10^{-10}
- Robust weighting: bisquare, cauchy, or, huber
- Parameter bounds: none

Step 6:

Compile the regression outputs, including the calculated viscosities vector, statistical performance indicators (R^2, RMSE, AARD), and parameter uncertainty estimates (Standard errors and confidence intervals).

The following tables represent the Intermediate Parameter estimations of the nonlinear regression

ChCl-MEA-W

Parameter	Estimate	Standard Error	95% CI
A_0	0.003908	0.0006292	0.002667–0.005149
E_0	21227	517.0	20207–22247
E_1	10933	397.0	10150–11716
a_{12}	4.5653	0.04337	4.4798–4.6508

ChCl-EG-W

Parameter	Estimate	SE	Approx. 95% CI
A_0	0.248	0.017454	0.1987–0.2674
E_0	4573.7	54.377	4422.7–4636.9
E_1	10746	191.15	10546.6–11299.4
a_{12}	2.5797	0.022247	2.5241–2.6117

ChCl-mCres-W

Parameter	Estimate	Standard Error	95% CI
A_0	0.0047276	0.0008967	0.002944–0.006511
E_0	10230	128.95	9973–10487
E_1	20094	476.23	19146–21042
a_{12}	2.7184	0.04874	2.621–2.815

ChCl-oCres-W

Parameter	Estimated value	SE	95% CI
A_0	0.005362	0.0023018	0.00085–0.01022
E_0	12448	376.98	11489–13025
E_1	19588	1041.6	17434–21678
a_{12}	2.988	0.1190	2.7166–3.2014

ChCl-1,2 Prop-W

Parameter	Estimate	Standard Error	95% Confidence Interval
A_0	0.01445	0.004281	0.006286–0.023344
E_0	8862.2	385.52	8092.9–9629.5
E_1	18338	730.13	16819–19731
a_{12}	3.727	0.09783	3.5311–3.9211

ChCl-1,3 Prop-W

Parameter	Estimate	Standard Error	95% CI
A_0	0.156	0.03055	0.09264–0.21440
E_0	3741.7	331.86	3051.4–4374.4
E_1	13573	507.13	12501–14723
a_{12}	3.718	0.07005	3.5744–3.8536

ChCl-Phenol-W

Parameter	Estimate	Standard Error	95% CI
A_0	0.021326	0.003884	0.01364–0.02902
E_0	10239	109.02	10021–10453
E_1	15856	456.47	14952–16760
a_{12}	3.1775	0.05823	3.0622–3.2928

ChCl-Glyc-W

Parameter	Estimate	Standard Error	95% CI
A_0	0.001749	0.00005718	0.001637–0.001861
E_0	4950.2	24.23	4902.6–4997.8
E_1	27833	47.63	27739–27927
a_{12}	3.7652	0.01047	3.7446–3.7858

S3: Experimental data used in this study as reported in the original sources

Tables S1, S2: Viscosity data of ChCl-MEA-W (Ref. [31, 32])

Tables S3, S4: Viscosity data of ChCl-EG-W (Ref. [34, 35])

Table S5: Viscosity data of ChCl-mCres-W (Ref. [28])

Table S6: Viscosity data of ChCl-oCres-W (Ref. [28])

Tables S7, S8: Viscosity data of ChCl-1,2 Prop-W (Ref. [28, 35])

Tables S9, S10: Viscosity data of ChCl-1,3 Prop-W (Ref. [28, 35])

Tables S11, S12: Viscosity data of ChCl-Phenol-W (Ref. [38, 39])

Tables S13, S14, S15, S16, S17: Viscosity data of ChCl-Glyc-W (Ref. [34, 36, 60–62])

Table S1 Viscosity data of ChCl-MEA-W (Ref. [31])

HBA:HBD molar ratio	water content W (wt%)	T (K)	η_{exp} (mPa·s)
1/6	0	293	55.62
		298	42.31
		313	20.57
		333	9.66
		353	5.38
	2.5	293	51
		298	38.99
		313	19.16
		333	9.02
		353	5.02
	5	293	48.8
		298	37.4
		313	18.7
		333	8.62
		353	4.83
	7.5	293	43.91
		298	34.42
		313	17.55
		333	8.06
		353	4.5
	10	293	41.23
		298	31.99
		313	15.83
		333	7.07
		353	4.11
	12.5	293	38.21
		298	29.1
		313	13.88
		333	6.98
		353	4
1/8	5	293	44.05
		298	33.92
		313	17.12
		333	7.78
		353	4.46
1/10	5	293	37.8
		298	29.22

		313	14.7
		333	7.19
		353	4.01

Table S2 Viscosity data of ChCl-MEA-W (Ref. [32])

HBA:HBD molar ratio	water content x (mol%)	T (K)	η_{exp} (mPa·s)
1/5	0	293.15	63.9
1/5	0	303.15	36.76
1/5	0	313.15	22.33
1/5	0	323.15	14.98
1/5	0.1009	293.15	58.42
1/5	0.1009	303.15	33.85
1/5	0.1009	313.15	20.75
1/5	0.1009	323.15	14.07
1/5	0.1999	293.15	51.4
1/5	0.1999	303.15	30.15
1/5	0.1999	313.15	18.68
1/5	0.1999	323.15	12.85
1/5	0.3001	293.15	43.27
1/5	0.3001	303.15	25.94
1/5	0.3001	313.15	16.3
1/5	0.3001	323.15	11.3
1/5	0.4	293.15	34.54
1/5	0.4	303.15	21.28
1/5	0.4	313.15	13.63
1/5	0.4	323.15	9.54
1/5	0.5037	293.15	25.87
1/5	0.5037	303.15	16.42
1/5	0.5037	313.15	10.73
1/5	0.5037	323.15	7.57
1/5	0.6017	293.15	18.23
1/5	0.6017	303.15	11.93
1/5	0.6017	313.15	7.95
1/5	0.6017	323.15	5.72
1/5	0.7018	293.15	11.53
1/5	0.7018	303.15	7.78
1/5	0.7018	313.15	5.31
1/5	0.7018	323.15	3.95
1/5	0.8	293.15	6.25
1/5	0.8	303.15	4.37

1/5	0.8	313.15	3.09
1/5	0.8	323.15	2.4
1/5	0.9006	293.15	2.57
1/5	0.9006	303.15	1.91
1/5	0.9006	313.15	1.41
1/5	0.9006	323.15	1.21
1/6	0	293.15	54.07
1/6	0	303.15	32.31
1/6	0	313.15	20.03
1/6	0	323.15	13.62
1/6	0.1018	293.15	50.14
1/6	0.1018	303.15	29.97
1/6	0.1018	313.15	18.69
1/6	0.1018	323.15	12.56
1/6	0.2002	293.15	45.29
1/6	0.2002	303.15	27.2
1/6	0.2002	313.15	17.05
1/6	0.2002	323.15	11.5
1/6	0.3	293.15	39.36
1/6	0.3	303.15	23.86
1/6	0.3	313.15	15.01
1/6	0.3	323.15	10.28
1/6	0.3999	293.15	32.71
1/6	0.3999	303.15	20.03
1/6	0.3999	313.15	12.7
1/6	0.3999	323.15	8.9
1/6	0.4999	293.15	25.63
1/6	0.4999	303.15	15.92
1/6	0.4999	313.15	10.15
1/6	0.4999	323.15	7.3
1/6	0.5997	293.15	18.49
1/6	0.5997	303.15	11.71
1/6	0.5997	313.15	7.61
1/6	0.5997	323.15	5.6
1/6	0.7	293.15	11.81
1/6	0.7	303.15	7.63
1/6	0.7	313.15	5.13
1/6	0.7	323.15	3.85
1/6	0.8001	293.15	6.3
1/6	0.8001	303.15	4.19
1/6	0.8001	313.15	2.98

1/6	0.8001	323.15	2.29
1/6	0.8998	293.15	2.45
1/6	0.8998	303.15	1.69
1/6	0.8998	313.15	1.41
1/6	0.8998	323.15	1.12
1/8	0	293.15	44.9
1/8	0	303.15	26.71
1/8	0	313.15	16.83
1/8	0	323.15	11.42
1/8	0.1	293.15	42.61
1/8	0.1	303.15	25.76
1/8	0.1	313.15	16.19
1/8	0.1	323.15	10.85
1/8	0.2246	293.15	38.55
1/8	0.2246	303.15	23.59
1/8	0.2246	313.15	14.9
1/8	0.2246	323.15	10.03
1/8	0.3008	293.15	35.32
1/8	0.3008	303.15	21.71
1/8	0.3008	313.15	13.79
1/8	0.3008	323.15	9.39
1/8	0.4	293.15	30.3
1/8	0.4	303.15	18.64
1/8	0.4	313.15	12.02
1/8	0.4	323.15	8.28
1/8	0.5	293.15	24.51
1/8	0.5	303.15	15.1
1/8	0.5	313.15	9.91
1/8	0.5	323.15	6.95
1/8	0.6	293.15	18.15
1/8	0.6	303.15	11.29
1/8	0.6	313.15	7.51
1/8	0.6	323.15	5.41
1/8	0.6999	293.15	11.88
1/8	0.6999	303.15	7.53
1/8	0.6999	313.15	5.09
1/8	0.6999	323.15	3.8
1/8	0.8	293.15	6.39
1/8	0.8	303.15	4.3
1/8	0.8	313.15	2.93
1/8	0.8	323.15	2.27

1/8	0.9	293.15	2.38
1/8	0.9	303.15	1.83
1/8	0.9	313.15	1.32
1/8	0.9	323.15	1.12
1/10	0	293.15	39.49
1/10	0	303.15	23.58
1/10	0	313.15	15.13
1/10	0	323.15	10.29
1/10	0.1001	293.15	37.94
1/10	0.1001	303.15	22.88
1/10	0.1001	313.15	14.61
1/10	0.1001	323.15	10.09
1/10	0.2001	293.15	35.92
1/10	0.2001	303.15	21.72
1/10	0.2001	313.15	13.92
1/10	0.2001	323.15	9.6
1/10	0.3206	293.15	32.07
1/10	0.3206	303.15	19.51
1/10	0.3206	313.15	12.6
1/10	0.3206	323.15	8.62
1/10	0.3999	293.15	28.63
1/10	0.3999	303.15	17.45
1/10	0.3999	313.15	11.38
1/10	0.3999	323.15	7.81
1/10	0.4998	293.15	23.3
1/10	0.4998	303.15	14.34
1/10	0.4998	313.15	9.47
1/10	0.4998	323.15	6.55
1/10	0.6	293.15	17.42
1/10	0.6	303.15	10.84
1/10	0.6	313.15	7.32
1/10	0.6	323.15	5.11
1/10	0.7	293.15	11.2
1/10	0.7	303.15	7.22
1/10	0.7	313.15	5.04
1/10	0.7	323.15	3.66
1/10	0.8001	293.15	5.66
1/10	0.8001	303.15	4
1/10	0.8001	313.15	2.95
1/10	0.8001	323.15	2.24
1/10	0.9	293.15	1.91

1/10	0.9	303.15	1.72
1/10	0.9	313.15	1.4
1/10	0.9	323.15	1.18

Table S3 Viscosity data of ChCl-EG-W (Ref. [34])

**HBA:HBD molar ratio**	**water content *x* (mol%)**	**T (K)**	**η_{exp} (mPa·s)**
1/2	1	288.15	1.14
1/2	1	293.15	1
1/2	1	298.15	0.89
1/2	1	303.15	0.8
1/2	1	308.15	0.72
1/2	1	313.15	0.65
1/2	1	318.15	0.6
1/2	1	323.15	0.55
1/2	0.99	288.15	1.25
1/2	0.99	293.15	1.09
1/2	0.99	298.15	0.97
1/2	0.99	303.15	0.87
1/2	0.99	308.15	0.79
1/2	0.99	313.15	0.71
1/2	0.99	318.15	0.66
1/2	0.99	323.15	0.6
1/2	0.98	288.15	1.33
1/2	0.98	293.15	1.17
1/2	0.98	298.15	1.04
1/2	0.98	303.15	0.93
1/2	0.98	308.15	0.84
1/2	0.98	313.15	0.76
1/2	0.98	318.15	0.71
1/2	0.98	323.15	0.65
1/2	0.97	288.15	1.45
1/2	0.97	293.15	1.28
1/2	0.97	298.15	1.14
1/2	0.97	303.15	1.02
1/2	0.97	308.15	0.92
1/2	0.97	313.15	0.83
1/2	0.97	318.15	0.76
1/2	0.97	323.15	0.71
1/2	0.96	288.15	1.56
1/2	0.96	293.15	1.39

1/2	0.96	298.15	1.23
1/2	0.96	303.15	1.1
1/2	0.96	308.15	0.99
1/2	0.96	313.15	0.9
1/2	0.96	318.15	0.81
1/2	0.96	323.15	0.75
1/2	0.95	288.15	1.69
1/2	0.95	293.15	1.48
1/2	0.95	298.15	1.33
1/2	0.95	303.15	1.19
1/2	0.95	308.15	1.07
1/2	0.95	313.15	0.97
1/2	0.95	318.15	0.88
1/2	0.95	323.15	0.8
1/2	0.94	288.15	1.83
1/2	0.94	293.15	1.6
1/2	0.94	298.15	1.42
1/2	0.94	303.15	1.28
1/2	0.94	308.15	1.15
1/2	0.94	313.15	1.04
1/2	0.94	318.15	0.94
1/2	0.94	323.15	0.86
1/2	0.93	288.15	1.97
1/2	0.93	293.15	1.72
1/2	0.93	298.15	1.52
1/2	0.93	303.15	1.37
1/2	0.93	308.15	1.23
1/2	0.93	313.15	1.11
1/2	0.93	318.15	1.01
1/2	0.93	323.15	0.92
1/2	0.9199	288.15	2.13
1/2	0.9199	293.15	1.86
1/2	0.9199	298.15	1.64
1/2	0.9199	303.15	1.46
1/2	0.9199	308.15	1.32
1/2	0.9199	313.15	1.19
1/2	0.9199	318.15	1.08
1/2	0.9199	323.15	0.98
1/2	0.9099	288.15	2.29
1/2	0.9099	293.15	1.99
1/2	0.9099	298.15	1.76

1/2	0.9099	303.15	1.56
1/2	0.9099	308.15	1.41
1/2	0.9099	313.15	1.27
1/2	0.9099	318.15	1.15
1/2	0.9099	323.15	1.05
1/2	0.9	288.15	2.46
1/2	0.9	293.15	2.14
1/2	0.9	298.15	1.88
1/2	0.9	303.15	1.67
1/2	0.9	308.15	1.49
1/2	0.9	313.15	1.36
1/2	0.9	318.15	1.24
1/2	0.9	323.15	1.12
1/2	0.7999	288.15	4.76
1/2	0.7999	293.15	4.09
1/2	0.7999	298.15	3.54
1/2	0.7999	303.15	3.1
1/2	0.7999	308.15	2.73
1/2	0.7999	313.15	2.42
1/2	0.7999	318.15	2.17
1/2	0.7999	323.15	1.95
1/2	0.7003	288.15	8.31
1/2	0.7003	293.15	7.03
1/2	0.7003	298.15	6.01
1/2	0.7003	303.15	5.17
1/2	0.7003	308.15	4.5
1/2	0.7003	313.15	3.95
1/2	0.7003	318.15	3.49
1/2	0.7003	323.15	3.1
1/2	0.6	288.15	13.43
1/2	0.6	293.15	11.19
1/2	0.6	298.15	9.42
1/2	0.6	303.15	8.02
1/2	0.6	308.15	6.89
1/2	0.6	313.15	5.98
1/2	0.6	318.15	5.23
1/2	0.6	323.15	4.61
1/2	0.5007	288.15	19
1/2	0.5007	293.15	15.65
1/2	0.5007	298.15	13.09
1/2	0.5007	303.15	11.06

1/2	0.5007	308.15	9.57
1/2	0.5007	313.15	8.16
1/2	0.5007	318.15	7.09
1/2	0.5007	323.15	6.21
1/2	0.3997	288.15	25.01
1/2	0.3997	293.15	20.38
1/2	0.3997	298.15	16.88
1/2	0.3997	303.15	14.23
1/2	0.3997	308.15	12.25
1/2	0.3997	313.15	10.43
1/2	0.3997	318.15	9.01
1/2	0.3997	323.15	7.9
1/2	0.3001	288.15	31.98
1/2	0.3001	293.15	25.86
1/2	0.3001	298.15	21.24
1/2	0.3001	303.15	17.67
1/2	0.3001	308.15	14.86
1/2	0.3001	313.15	12.98
1/2	0.3001	318.15	11.38
1/2	0.3001	323.15	9.98
1/2	0.2001	288.15	42.29
1/2	0.2001	293.15	33.92
1/2	0.2001	298.15	27.65
1/2	0.2001	303.15	22.85
1/2	0.2001	308.15	19.1
1/2	0.2001	313.15	16.15
1/2	0.2001	318.15	13.95
1/2	0.2001	323.15	12.32
1/2	0.1001	288.15	54.67
1/2	0.1001	293.15	43.67
1/2	0.1001	298.15	35.36
1/2	0.1001	303.15	28.98
1/2	0.1001	308.15	24.16
1/2	0.1001	313.15	20.27
1/2	0.1001	318.15	17.18
1/2	0.1001	323.15	14.76
1/2	0	288.15	67.04
1/2	0	293.15	53.07
1/2	0	298.15	43.03
1/2	0	303.15	35.06
1/2	0	308.15	29.02

1/2	0	313.15	24.22
1/2	0	318.15	20.47
1/2	0	323.15	17.41

Table S4 Viscosity data of ChCl-EG-W (Ref. [35])

HBA:HBD molar ratio	water content x (mol%)	T (K)	η_{exp} (mPa·s)
1/6	0	293.15	27.44
1/6	0	303.15	19.43
1/6	0	313.15	14.16
1/6	0	323.15	11.09
1/6	0	333.15	8.46
1/6	0.05	293.15	25.93
1/6	0.05	303.15	17.74
1/6	0.05	313.15	12.78
1/6	0.05	323.15	9.38
1/6	0.05	333.15	6.86
1/6	0.1	293.15	24.39
1/6	0.1	303.15	16.71
1/6	0.1	313.15	12.17
1/6	0.1	323.15	8.38
1/6	0.1	333.15	6.39
1/6	0.3	293.15	18.01
1/6	0.3	303.15	12.56
1/6	0.3	313.15	9.15
1/6	0.3	323.15	6.4
1/6	0.3	333.15	4.93
1/6	0.5	293.15	10.79
1/6	0.5	303.15	7.61
1/6	0.5	313.15	5.59
1/6	0.5	323.15	4.25
1/6	0.5	333.15	3.33
1/6	0.7	293.15	5.58
1/6	0.7	303.15	4.08
1/6	0.7	313.15	3.1
1/6	0.7	323.15	2.43
1/6	0.7	333.15	1.95
1/6	0.9	293.15	2.1
1/6	0.9	303.15	1.61
1/6	0.9	313.15	1.28
1/6	0.9	323.15	1.04

1/6	0.9	333.15	0.87
1/6	0.95	293.15	1.55
1/6	0.95	303.15	1.2
1/6	0.95	313.15	0.97
1/6	0.95	323.15	0.79
1/6	0.95	333.15	0.67
1/3	0	293.15	37.35
1/3	0	303.15	25.72
1/3	0	313.15	18.27
1/3	0	323.15	13.57
1/3	0	333.15	10.47
1/3	0.05	293.15	36.51
1/3	0.05	303.15	24.78
1/3	0.05	313.15	17.7
1/3	0.05	323.15	13.18
1/3	0.05	333.15	9.85
1/3	0.1	293.15	33.98
1/3	0.1	303.15	23.12
1/3	0.1	313.15	16.53
1/3	0.1	323.15	12.43
1/3	0.1	333.15	8.93
1/3	0.3	293.15	23.85
1/3	0.3	303.15	16.45
1/3	0.3	313.15	11.25
1/3	0.3	323.15	8.39
1/3	0.3	333.15	6.77
1/3	0.5	293.15	13.24
1/3	0.5	303.15	9.34
1/3	0.5	313.15	6.87
1/3	0.5	323.15	5.22
1/3	0.5	333.15	4.09
1/3	0.7	293.15	6.39
1/3	0.7	303.15	4.69
1/3	0.7	313.15	3.57
1/3	0.7	323.15	2.8
1/3	0.7	333.15	2.25
1/3	0.9	293.15	2.13
1/3	0.9	303.15	1.65
1/3	0.9	313.15	1.32
1/3	0.9	323.15	1.08
1/3	0.9	333.15	0.91

1/3	0.95	293.15	1.5
1/3	0.95	303.15	1.18
1/3	0.95	313.15	0.95
1/3	0.95	323.15	0.79
1/3	0.95	333.15	0.67
0	1	293.15	1.002
0	1	303.15	0.797
0	1	313.15	0.653
0	1	323.15	0.547
0	1	333.15	0.466

Table S5 Viscosity data of ChCl-mCres-W (Ref. [28])

HBA:HBD molar ratio	water content x (mol%)	T (K)	η_{exp} (mPa·s)
1/2	0.25	283	121.17
1/2	0.25	293	62
1/2	0.25	303	35.73
1/2	0.25	313	23.77
1/2	0.25	323	16.95
1/2	0.25	333	12.78
1/2	0.42	283	62.75
1/2	0.42	293	33.05
1/2	0.42	303	20.85
1/2	0.42	313	14.15
1/2	0.42	323	10.65
1/2	0.42	333	8.5
1/2	0.54	283	38.5
1/2	0.54	293	21.2
1/2	0.54	303	14.2
1/2	0.54	313	10.02
1/2	0.54	323	7.74
1/2	0.54	333	6.46
1/3	0.25	283	78.6
1/3	0.25	293	41.8
1/3	0.25	303	25.88
1/3	0.25	313	17.65
1/3	0.25	323	12.82
1/3	0.25	333	9.99
1/3	0.41	283	42.15
1/3	0.41	293	23.08
1/3	0.41	303	14.37

1/3	0.41	313	10.01
1/3	0.41	323	7.31
1/3	0.41	333	5.64
1/3	0.53	283	26.72
1/3	0.53	293	15.5
1/3	0.53	303	10.43
1/3	0.53	313	7.61
1/3	0.53	323	5.75
1/3	0.53	333	4.51
1/5	0.25	283	48.12
1/5	0.25	293	26.62
1/5	0.25	303	16.35
1/5	0.25	313	11.2
1/5	0.25	323	8.13
1/5	0.25	333	6.16
1/5	0.41	283	28.52
1/5	0.41	293	15.85
1/5	0.41	303	10.08
1/5	0.41	313	7.24
1/5	0.41	323	5.39
1/5	0.41	333	4.31
1/5	0.52	283	23.2
1/5	0.52	293	13.53
1/5	0.52	303	8.95
1/5	0.52	313	6.42
1/5	0.52	323	4.87
1/5	0.52	333	3.84
1/2	0	293	155
1/2	0	298	111
1/2	0	303	81.8
1/2	0	308	62.1
1/2	0	313	48.5
1/2	0	318	38.7
1/2	0	323	31
1/2	0	333	21.1
1/3	0	293	74.7
1/3	0	298	55
1/3	0	303	42.6
1/3	0	308	33.6
1/3	0	313	27.5
1/3	0	318	21.5

1/3	0	323	18.2
1/3	0	333	13
1/4	0	293	49.7
1/4	0	298	37.3
1/4	0	303	29.2
1/4	0	308	23.4
1/4	0	313	18.9
1/4	0	318	15.8
1/4	0	323	13.3
1/4	0	333	9.92
1/5	0	293	41.1
1/5	0	298	30.5
1/5	0	303	24.7
1/5	0	308	19.1
1/5	0	313	15.9
1/5	0	318	12.7
1/5	0	323	10.8
1/5	0	333	7.73

Table S6 Viscosity data of ChCl-oCres-W (Ref. [28])

HBA:HBD molar ratio	water content *x* (mol%)	T (K)	η_{exp} (mPa·s)
1/3	0.68	283	18.4
1/3	0.68	293	11.2
1/3	0.68	303	7.96
1/3	0.68	313	6.01
1/3	0.68	323	4.87
1/3	0.68	333	3.94
1/3	0.53	283	34.1
1/3	0.53	293	19.3
1/3	0.53	303	12.4
1/3	0.53	313	8.74
1/3	0.53	323	6.67
1/3	0.53	333	5.29
1/3	0.26	283	85.63
1/3	0.26	293	48.7
1/3	0.26	303	28.2
1/3	0.26	313	18.4
1/3	0.26	323	12.9
1/3	0.26	333	9.56
2/5	0	293	124.67

2/5	0	298	89.53
2/5	0	303	66.73
2/5	0	308	53.27
2/5	0	313	40.77
2/5	0	318	37.92
1/4	0	293	60.9
1/4	0	298	45.22
1/4	0	303	34.9
1/4	0	308	28.27
1/4	0	313	22.5
1/4	0	318	19.3
1/5	0	293	46.17
1/5	0	298	34.92
1/5	0	303	27.65
1/5	0	308	22.48
1/5	0	313	18.68
1/5	0	318	15.82

Table S7 Viscosity data of ChCl-1,2 Prop-W (Ref. [28])

HBA:HBD molar ratio	water content *x* (mol%)	T (K)	η_{exp} (mPa·s)
1/3	0.63	283	28.08
1/3	0.63	293	17.13
1/3	0.63	303	11.72
1/3	0.63	313	8.7
1/3	0.63	323	6.64
1/3	0.63	333	5.36
1/3	0.48	283	48.72
1/3	0.48	293	27.02
1/3	0.48	303	17.05
1/3	0.48	313	11.58
1/3	0.48	323	9.28
1/3	0.48	333	7.33
1/3	0.21	283	107
1/3	0.21	293	57.9
1/3	0.21	303	34.62
1/3	0.21	313	22.18
1/3	0.21	323	16.12
1/3	0.21	333	11.55
1/4	0	293	64.3
1/4	0	298	47.8

1/4	0	303	36.8
1/4	0	308	29.7
1/4	0	313	25.3
1/4	0	318	22.8
1/6	0	293	52.6
1/6	0	298	40.5
1/6	0	303	31
1/6	0	308	24.8
1/6	0	313	19.9
1/6	0	318	17.3
1/7	0	293	49.2
1/7	0	298	35.2
1/7	0	303	26.4
1/7	0	308	21.6
1/7	0	313	17.9
1/7	0	318	15.2

Table S8 Viscosity data of ChCl-1,2 Prop-W (Ref. [35])

HBA:HBD molar ratio	water content x (mol%)	T (K)	η_{exp} (mPa·s)
1/3	0	293.15	94.05
1/3	0	303.15	56.26
1/3	0	313.15	35.89
1/3	0	323.15	24.25
1/3	0	333.15	17.11
1/3	0.05	293.15	87.34
1/3	0.05	303.15	52.34
1/3	0.05	313.15	33.12
1/3	0.05	323.15	22.33
1/3	0.05	333.15	15.79
1/3	0.1	293.15	80.67
1/3	0.1	303.15	48.25
1/3	0.1	313.15	31
1/3	0.1	323.15	20.68
1/3	0.1	333.15	14.65
1/3	0.3	293.15	52.76
1/3	0.3	303.15	32.37
1/3	0.3	313.15	21.25
1/3	0.3	323.15	14.15
1/3	0.3	333.15	10.17
1/3	0.5	293.15	31.29

1/3	0.5	303.15	19.75
1/3	0.5	313.15	13.36
1/3	0.5	323.15	8.91
1/3	0.5	333.15	6.79
1/3	0.7	293.15	12.18
1/3	0.7	303.15	8.16
1/3	0.7	313.15	5.75
1/3	0.7	323.15	4.23
1/3	0.7	333.15	3.26
1/3	0.9	293.15	3.04
1/3	0.9	303.15	2.24
1/3	0.9	313.15	1.72
1/3	0.9	323.15	1.36
1/3	0.9	333.15	1.11
1/3	0.95	293.15	1.83
1/3	0.95	303.15	1.4
1/3	0.95	313.15	1.11
1/3	0.95	323.15	0.9
1/3	0.95	333.15	0.75

Table S9 Viscosity data of ChCl-1,3 Prop-W (Ref. [28])

HBA:HBD molar ratio	water content x (mol%)	T (K)	η_{exp} (mPa·s)
1/3	0.63	283	21.92
1/3	0.63	293	13.23
1/3	0.63	303	8.89
1/3	0.63	313	6.66
1/3	0.63	323	5.42
1/3	0.63	333	4.93
1/3	0.47	283	39.52
1/3	0.47	293	23.48
1/3	0.47	303	14.57
1/3	0.47	313	11.1
1/3	0.47	323	8.17
1/3	0.47	333	6.75
1/3	0.21	283	81.47
1/3	0.21	293	40.78
1/3	0.21	303	30.48
1/3	0.21	313	20.47
1/3	0.21	323	15.65
1/3	0.21	333	14.27

1/3	0	293	68.33
1/3	0	298	50.72
1/3	0	303	42.48
1/3	0	308	37.15
1/3	0	313	30.82
1/3	0	318	26.52
1/4	0	293	60
1/4	0	298	47.75
1/4	0	303	38.78
1/4	0	308	32.47
1/4	0	313	26.48
1/4	0	318	22.48
1/5	0	293	56
1/5	0	298	44.4
1/5	0	303	37.1
1/5	0	308	30.6
1/5	0	313	25.4
1/5	0	318	21.6

Table S10 Viscosity data of ChCl-1,3 Prop-W (Ref. [35])

HBA:HBD molar ratio	water content *x* (mol%)	T (K)	η_{exp} (mPa·s)
1/3	0	293.15	69.74
1/3	0	303.15	45.4
1/3	0	313.15	31.35
1/3	0	323.15	22.62
1/3	0	333.15	16.88
1/3	0.05	293.15	66
1/3	0.05	303.15	43.17
1/3	0.05	313.15	29.71
1/3	0.05	323.15	20.95
1/3	0.05	333.15	15.52
1/3	0.1	293.15	61.22
1/3	0.1	303.15	40.2
1/3	0.1	313.15	27.31
1/3	0.1	323.15	19.56
1/3	0.1	333.15	14.49
1/3	0.3	293.15	43.4
1/3	0.3	303.15	28.73
1/3	0.3	313.15	20.04
1/3	0.3	323.15	14.14

1/3	0.3	333.15	10.53
1/3	0.5	293.15	27.3
1/3	0.5	303.15	18.39
1/3	0.5	313.15	13
1/3	0.5	323.15	8.96
1/3	0.5	333.15	6.75
1/3	0.7	293.15	12.15
1/3	0.7	303.15	8.21
1/3	0.7	313.15	5.59
1/3	0.7	323.15	4.22
1/3	0.7	333.15	3.28
1/3	0.9	293.15	2.8
1/3	0.9	303.15	2.11
1/3	0.9	313.15	1.65
1/3	0.9	323.15	1.32
1/3	0.9	333.15	1.12
1/3	0.95	293.15	1.75
1/3	0.95	303.15	1.36
1/3	0.95	313.15	1.08
1/3	0.95	323.15	0.89
1/3	0.95	333.15	0.75

Table S11 Viscosity data of ChCl-Phenol-W (Ref. [38])

HBA:HBD molar ratio	water content x (mol%)	T (K)	η_{exp} (mPa·s)
1/2	0	293.2	120.77
1/2	0	298.2	90.33
1/2	0	303.2	68.41
1/2	0	308.2	53.43
1/2	0	313.2	42.42
1/2	0	318.2	34.34
1/3	0	293.2	57.84
1/3	0	298.2	44.64
1/3	0	303.2	35.17
1/3	0	308.2	28.22
1/3	0	313.2	23.08
1/3	0	318.2	19.14
1/4	0	293.2	40.23
1/4	0	298.2	31.55
1/4	0	303.2	25.2
1/4	0	308.2	20.25

1/4	0	313.2	16.71
1/4	0	318.2	14
1/5	0	293.2	31.96
1/5	0	298.2	25.25
1/5	0	303.2	19.75
1/5	0	308.2	16.16
1/5	0	313.2	13.44
1/5	0	318.2	11.26
1/6	0	293.2	27.03
1/6	0	298.2	21.43
1/6	0	303.2	16.82
1/6	0	308.2	13.76
1/6	0	313.2	11.45
1/6	0	318.2	9.46

Table S12 Viscosity data of ChCl-Phenol-W (Ref. [39])

HBA:HBD molar ratio	water content x (mol%)	T (K)	η_{exp} (mPa·s)
1/3	0	323.15	19.109
1/3	0	333.15	13.769
1/3	0.1	293.15	56.094
1/3	0.1	303.15	34.531
1/3	0.1	313.15	22.761
1/3	0.1	323.15	15.893
1/3	0.1	333.15	11.618
1/3	0.2	293.15	40.935
1/3	0.2	303.15	26.614
1/3	0.2	313.15	17.593
1/3	0.2	323.15	12.403
1/3	0.2	333.15	9.216
1/3	0.3	293.15	29.561
1/3	0.3	303.15	19.262
1/3	0.3	313.15	13.304
1/3	0.3	323.15	9.647
1/3	0.3	333.15	7.279
1/3	0.401	293.15	21.484
1/3	0.401	303.15	14.376
1/3	0.401	313.15	10.142
1/3	0.401	323.15	7.48
1/3	0.401	333.15	5.722
1/3	0.499	293.15	15.369

1/3	0.499	303.15	10.554
1/3	0.499	313.15	7.612
1/3	0.499	323.15	5.716
1/3	0.499	333.15	4.437
1/3	0.601	293.15	10.567
1/3	0.601	303.15	7.469
1/3	0.601	313.15	5.522
1/3	0.601	323.15	4.232
1/3	0.601	333.15	3.34
1/3	0.7	293.15	7.478
1/3	0.7	303.15	5.415
1/3	0.7	313.15	4.077
1/3	0.7	323.15	3.175
1/3	0.7	333.15	2.542
1/3	0.8	293.15	4.918
1/3	0.8	303.15	3.639
1/3	0.8	313.15	2.792
1/3	0.8	323.15	2.209
1/3	0.8	333.15	1.794
1/3	0.9	293.15	2.774
1/3	0.9	303.15	2.094
1/3	0.9	313.15	1.639
1/3	0.9	323.15	1.32
1/3	0.9	333.15	1.089
1/4	0.1	293.15	43.9
1/4	0.1	303.15	27.667
1/4	0.1	313.15	18.552
1/4	0.1	323.15	13.26
1/4	0.1	333.15	9.923
1/4	0.2	293.15	34.948
1/4	0.2	303.15	22.499
1/4	0.2	313.15	15.237
1/4	0.2	323.15	11.035
1/4	0.2	333.15	8.309
1/4	0.3	293.15	27.379
1/4	0.3	303.15	17.929
1/4	0.3	313.15	12.394
1/4	0.3	323.15	9.002
1/4	0.3	333.15	6.8
1/4	0.4	293.15	20.064
1/4	0.4	303.15	13.501

1/4	0.4	313.15	9.539
1/4	0.4	323.15	7.046
1/4	0.4	333.15	5.391
1/4	0.5	293.15	14.906
1/4	0.5	303.15	10.306
1/4	0.5	313.15	7.438
1/4	0.5	323.15	5.586
1/4	0.5	333.15	4.334
1/4	0.6	293.15	11.076
1/4	0.6	303.15	7.782
1/4	0.6	313.15	5.722
1/4	0.6	323.15	4.364
1/4	0.6	333.15	3.433
1/4	0.701	293.15	7.315
1/4	0.701	303.15	5.29
1/4	0.701	313.15	3.983
1/4	0.701	323.15	3.101
1/4	0.701	333.15	2.485
1/4	0.8	293.15	4.816
1/4	0.8	303.15	3.576
1/4	0.8	313.15	2.739
1/4	0.8	323.15	2.163
1/4	0.8	333.15	1.753
1/4	0.9	293.15	2.827
1/4	0.9	303.15	2.128
1/4	0.9	313.15	1.66
1/4	0.9	323.15	1.334
1/4	0.9	333.15	1.099

Table S13 Viscosity data of ChCl-Glyc-W (Ref. [34])

HBA:HBD molar ratio	water content *x* (mol%)	T (K)	η_{exp} (mPa·s)
1/2	0.99	288.15	1.28
1/2	0.99	293.15	1.12
1/2	0.99	298.15	1
1/2	0.99	303.15	0.9
1/2	0.99	308.15	0.81
1/2	0.99	313.15	0.73
1/2	0.99	318.15	0.67
1/2	0.99	323.15	0.63
1/2	0.98	288.15	1.43

1/2	0.98	293.15	1.26
1/2	0.98	298.15	1.12
1/2	0.98	303.15	1
1/2	0.98	308.15	0.9
1/2	0.98	313.15	0.82
1/2	0.98	318.15	0.74
1/2	0.98	323.15	0.68
1/2	0.97	288.15	1.6
1/2	0.97	293.15	1.41
1/2	0.97	298.15	1.25
1/2	0.97	303.15	1.11
1/2	0.97	308.15	1
1/2	0.97	313.15	0.9
1/2	0.97	318.15	0.82
1/2	0.97	323.15	0.75
1/2	0.96	288.15	1.78
1/2	0.96	293.15	1.56
1/2	0.96	298.15	1.38
1/2	0.96	303.15	1.23
1/2	0.96	308.15	1.1
1/2	0.96	313.15	1
1/2	0.96	318.15	0.9
1/2	0.96	323.15	0.83
1/2	0.9501	288.15	1.98
1/2	0.9501	293.15	1.73
1/2	0.9501	298.15	1.52
1/2	0.9501	303.15	1.36
1/2	0.9501	308.15	1.22
1/2	0.9501	313.15	1.1
1/2	0.9501	318.15	1
1/2	0.9501	323.15	0.91
1/2	0.9401	288.15	2.2
1/2	0.9401	293.15	1.92
1/2	0.9401	298.15	1.69
1/2	0.9401	303.15	1.49
1/2	0.9401	308.15	1.34
1/2	0.9401	313.15	1.21
1/2	0.9401	318.15	1.09
1/2	0.9401	323.15	1
1/2	0.9302	288.15	2.44
1/2	0.9302	293.15	2.12

1/2	0.9302	298.15	1.86
1/2	0.9302	303.15	1.65
1/2	0.9302	308.15	1.47
1/2	0.9302	313.15	1.33
1/2	0.9302	318.15	1.2
1/2	0.9302	323.15	1.09
1/2	0.9201	288.15	2.66
1/2	0.9201	293.15	2.32
1/2	0.9201	298.15	2.04
1/2	0.9201	303.15	1.81
1/2	0.9201	308.15	1.61
1/2	0.9201	313.15	1.45
1/2	0.9201	318.15	1.32
1/2	0.9201	323.15	1.2
1/2	0.9101	288.15	3.11
1/2	0.9101	293.15	2.69
1/2	0.9101	298.15	2.35
1/2	0.9101	303.15	2.06
1/2	0.9101	308.15	1.84
1/2	0.9101	313.15	1.64
1/2	0.9101	318.15	1.47
1/2	0.9101	323.15	1.34
1/2	0.9	288.15	3.29
1/2	0.9	293.15	2.84
1/2	0.9	298.15	2.47
1/2	0.9	303.15	2.17
1/2	0.9	308.15	1.92
1/2	0.9	313.15	1.72
1/2	0.9	318.15	1.54
1/2	0.9	323.15	1.4
1/2	0.8004	288.15	8.8
1/2	0.8004	293.15	7.34
1/2	0.8004	298.15	6.19
1/2	0.8004	303.15	5.28
1/2	0.8004	308.15	4.55
1/2	0.8004	313.15	3.96
1/2	0.8004	318.15	3.48
1/2	0.8004	323.15	3.07
1/2	0.7001	288.15	17.71
1/2	0.7001	293.15	14.33
1/2	0.7001	298.15	12.16

1/2	0.7001	303.15	10.28
1/2	0.7001	308.15	8.72
1/2	0.7001	313.15	7.41
1/2	0.7001	318.15	6.31
1/2	0.7001	323.15	5.37
1/2	0.6	288.15	36.8
1/2	0.6	293.15	28.68
1/2	0.6	298.15	22.75
1/2	0.6	303.15	18.36
1/2	0.6	308.15	15.03
1/2	0.6	313.15	12.82
1/2	0.6	318.15	11.01
1/2	0.6	323.15	9.46
1/2	0.5001	288.15	68.67
1/2	0.5001	293.15	51.92
1/2	0.5001	298.15	40.21
1/2	0.5001	303.15	31.66
1/2	0.5001	308.15	25.33
1/2	0.5001	313.15	20.61
1/2	0.5001	318.15	16.94
1/2	0.5001	323.15	14.2
1/2	0.4	288.15	120.04
1/2	0.4	293.15	88.51
1/2	0.4	298.15	66.51
1/2	0.4	303.15	51.16
1/2	0.4	308.15	40.42
1/2	0.4	313.15	32.24
1/2	0.4	318.15	26.09
1/2	0.4	323.15	21.4
1/2	0.3001	288.15	199.14
1/2	0.3001	293.15	141.84
1/2	0.3001	298.15	104.94
1/2	0.3001	303.15	79.09
1/2	0.3001	308.15	60.97
1/2	0.3001	313.15	47.78
1/2	0.3001	318.15	38.4
1/2	0.3001	323.15	31.05
1/2	0.1999	288.15	372.4
1/2	0.1999	293.15	265.3
1/2	0.1999	298.15	192.52
1/2	0.1999	303.15	143.54

1/2	0.1999	308.15	108.98
1/2	0.1999	313.15	83.86
1/2	0.1999	318.15	66.5
1/2	0.1999	323.15	52.58
1/2	0.1003	288.15	551.58
1/2	0.1003	293.15	385.8
1/2	0.1003	298.15	278.35
1/2	0.1003	303.15	203.71
1/2	0.1003	308.15	152.74
1/2	0.1003	313.15	116.8
1/2	0.1003	318.15	90.32
1/2	0.1003	323.15	71.21
1/2	0	288.15	749.16
1/2	0	293.15	516.05
1/2	0	298.15	364.14
1/2	0	303.15	265.86
1/2	0	308.15	194.55
1/2	0	313.15	148.61
1/2	0	318.15	114.36
1/2	0	323.15	88.65

Table S14 Viscosity data of ChCl-Glyc-W (Ref. [36])

HBA:HBD molar ratio	water content x (mol%)	T (K)	η_{exp} (mPa·s)
1/2	0.108	283.15	669.9038
1/2	0.108	333.15	39.3459
1/2	0.108	343.15	27.497
1/2	0.108	353.15	20.0963
1/2	0.108	363.15	15.2351
1/2	0.2381	283.15	397.1261
1/2	0.2381	333.15	28.2346
1/2	0.2381	343.15	20.1102
1/2	0.2381	353.15	14.9828
1/2	0.2381	363.15	11.4031
1/2	0.3425	283.15	239.0806
1/2	0.3425	333.15	20.8106
1/2	0.3425	343.15	15.2347
1/2	0.3425	353.15	11.586
1/2	0.3425	363.15	9.0466
1/2	0.4022	283.15	177.9621
1/2	0.4022	333.15	17.1371

1/2	0.4022	343.15	12.6879
1/2	0.4022	353.15	9.5736
1/2	0.4022	363.15	7.5048
1/2	0.5978	283.15	54.6306
1/2	0.5978	333.15	7.7082
1/2	0.5978	343.15	5.6748
1/2	0.5978	353.15	4.4946
1/2	0.5978	363.15	3.6385

Table S15 Viscosity data of ChCl-Glyc-W (Ref. [60])

HBA:HBD molar ratio	water content x (mol%)	T (K)	η_{exp} (mPa·s)
1/3	0.39066	298.15	62.045
1/3	0.39066	303.15	52.91
1/3	0.39066	308.15	41.885
1/3	0.39066	313.15	33.395
1/3	0.39066	318.15	26.48
1/3	0.39066	323.15	21.15
1/3	0.39066	328.15	18.76
1/3	0.39066	333.15	14.45
1/3	0.39066	338.15	12.385
1/3	0.39066	343.15	10.975

Table S16 Viscosity data of ChCl-Glyc-W (Ref. [61])

HBA:HBD molar ratio	water content x (mol%)	T (K)	η_{exp} (mPa·s)
1/2	0.02	294.13	397.22
1/2	0.02	295.17	373.88
1/2	0.02	296.18	349.7
1/2	0.02	297.19	327.07
1/2	0.02	298.2	306.15
1/2	0.02	299.2	286.07
1/2	0.02	300.2	268.15
1/2	0.02	301.2	250.95
1/2	0.02	302.2	235.31
1/2	0.02	303.2	220.96
1/2	0.02	304.2	207.8
1/2	0.02	305.2	195.77
1/2	0.02	306.2	184.24
1/2	0.02	307.2	173.7

1/2	0.02	308.2	163.83
1/2	0.02	309.2	154.58
1/2	0.02	310.2	146.05
1/2	0.02	311.2	138.16
1/2	0.02	312.2	130.74
1/2	0.02	313.2	123.91
1/2	0.02	314.2	117.46
1/2	0.02	315.2	111.4
1/2	0.02	316.2	105.65
1/2	0.02	317.2	100.55
1/2	0.02	318.2	95.65
1/2	0.02	319.2	90.96
1/2	0.02	320.2	86.61
1/2	0.02	321.2	82.5
1/2	0.02	322.2	78.76
1/2	0.02	323.2	75.17
1/2	0.16	294.13	228.4
1/2	0.16	295.17	215.19
1/2	0.16	296.18	203.08
1/2	0.16	297.19	191.9
1/2	0.16	298.2	182.46
1/2	0.16	299.2	174.23
1/2	0.16	300.2	166.13
1/2	0.16	301.2	154.96
1/2	0.16	302.2	152.52
1/2	0.16	303.2	140.59
1/2	0.16	304.2	135.54
1/2	0.16	305.2	126.81
1/2	0.16	306.2	123.68
1/2	0.16	307.2	116.5
1/2	0.16	308.2	112.09
1/2	0.16	309.2	107.19
1/2	0.16	310.2	99.3
1/2	0.16	311.2	97.25
1/2	0.16	312.2	91.09
1/2	0.16	313.2	86.87
1/2	0.16	314.2	83.11
1/2	0.16	315.2	78.71
1/2	0.16	316.2	75.61
1/2	0.16	317.2	72.35
1/2	0.16	318.2	70.37

1/2	0.16	319.2	67.93
1/2	0.16	320.2	68.75
1/2	0.16	321.2	64.26
1/2	0.16	322.2	65.67
1/2	0.16	323.2	65.27
1/2	0.42	294.13	82.07
1/2	0.42	295.17	79.57
1/2	0.42	296.18	75.69
1/2	0.42	297.19	71.85
1/2	0.42	298.2	68.13
1/2	0.42	299.2	64.65
1/2	0.42	300.2	61.4
1/2	0.42	301.2	58.4
1/2	0.42	302.2	55.55
1/2	0.42	303.2	52.94
1/2	0.42	304.2	50.53
1/2	0.42	305.2	48.3
1/2	0.42	306.2	46.15
1/2	0.42	307.2	44.08
1/2	0.42	308.2	42.24
1/2	0.42	309.2	40.49
1/2	0.42	310.2	38.84
1/2	0.42	311.2	37.33
1/2	0.42	312.2	35.78
1/2	0.42	313.2	34.39
1/2	0.42	314.2	33.11
1/2	0.42	315.2	31.93
1/2	0.42	316.2	30.73
1/2	0.42	317.2	29.63
1/2	0.42	318.2	28.6
1/2	0.42	319.2	27.6
1/2	0.42	320.2	26.63
1/2	0.42	321.2	25.77
1/2	0.42	322.2	24.9
1/2	0.42	323.2	24.17
1/2	0.6	294.13	31.03
1/2	0.6	295.17	29.66
1/2	0.6	296.18	28.39
1/2	0.6	297.19	27.15
1/2	0.6	298.2	25.96
1/2	0.6	299.2	24.87

1/2	0.6	300.2	23.84
1/2	0.6	301.2	22.9
1/2	0.6	302.2	21.99
1/2	0.6	303.2	21.14
1/2	0.6	304.2	20.36
1/2	0.6	305.2	19.6
1/2	0.6	306.2	18.91
1/2	0.6	307.2	18.23
1/2	0.6	308.2	17.62
1/2	0.6	309.2	17.03
1/2	0.6	310.2	16.49
1/2	0.6	311.2	15.96
1/2	0.6	312.2	15.44
1/2	0.6	313.2	14.97
1/2	0.6	314.2	14.53
1/2	0.6	315.2	14.09
1/2	0.6	316.2	13.71
1/2	0.6	317.2	13.34
1/2	0.6	318.2	13.01
1/2	0.6	319.2	12.66
1/2	0.6	320.2	12.31
1/2	0.6	321.2	12.02
1/2	0.6	322.2	11.71
1/2	0.6	323.2	11.45
1/2	0.76	294.13	8.43
1/2	0.76	295.17	8.13
1/2	0.76	296.18	7.86
1/2	0.76	297.19	7.6
1/2	0.76	298.2	7.34
1/2	0.76	299.2	7.1
1/2	0.76	300.2	6.88
1/2	0.76	301.2	6.69
1/2	0.76	302.2	6.49
1/2	0.76	303.2	6.32
1/2	0.76	304.2	6.13
1/2	0.76	305.2	5.96
1/2	0.76	306.2	5.78
1/2	0.76	307.2	5.64
1/2	0.76	308.2	5.49
1/2	0.76	309.2	5.36
1/2	0.76	310.2	5.24

1/2	0.76	311.2	5.13
1/2	0.76	312.2	5.01
1/2	0.76	313.2	4.87
1/2	0.76	314.2	4.76
1/2	0.76	315.2	4.65
1/2	0.76	316.2	4.56
1/2	0.76	317.2	4.46
1/2	0.76	318.2	4.39
1/2	0.76	319.2	4.31
1/2	0.76	320.2	4.21
1/2	0.76	321.2	4.13
1/2	0.76	322.2	4.04
1/2	0.76	323.2	3.96

Table S17 Viscosity data of ChCl-Glyc-W (Ref. [62])

HBA:HBD molar ratio	water content x (mol%)	T (K)	η_{exp} (mPa·s)
1/2	0	278.15	1511
1/2	0	280.65	1211
1/2	0	283.15	982.4
1/2	0	285.65	801.5
1/2	0	288.15	658.4
1/2	0	290.65	543.7
1/2	0	293.15	452.6
1/2	0	295.65	378.5
1/2	0	298.15	319.7
1/2	0	300.65	271.4
1/2	0	303.15	232.1
1/2	0	305.65	199
1/2	0	308.15	171.6
1/2	0	310.65	149
1/2	0	313.15	130
1/2	0	315.65	113.8
1/2	0	318.15	100
1/2	0	320.65	88.43
1/2	0	323.15	78.39
1/2	0	325.65	69.46
1/2	0	328.15	62.06
1/2	0	330.65	55.63
1/2	0	333.15	50.05
1/2	0	335.65	45.19

1/2	0	338.15	40.94
1/2	0.255	278.15	594
1/2	0.255	280.65	488.1
1/2	0.255	283.15	404.4
1/2	0.255	285.65	337
1/2	0.255	288.15	282.3
1/2	0.255	290.65	238.6
1/2	0.255	293.15	203
1/2	0.255	295.65	172.6
1/2	0.255	298.15	147.8
1/2	0.255	300.65	128
1/2	0.255	303.15	111.3
1/2	0.255	305.65	97.13
1/2	0.255	308.15	85.25
1/2	0.255	310.65	75.14
1/2	0.255	313.15	66.48
1/2	0.255	315.65	59.01
1/2	0.255	318.15	52.61
1/2	0.255	320.65	47.12
1/2	0.255	323.15	42.48
1/2	0.255	325.65	38.28
1/2	0.255	328.15	34.66
1/2	0.255	330.65	31.47
1/2	0.255	333.15	28.66
1/2	0.255	335.65	26.14
1/2	0.255	338.15	23.94
